# mRNA lipid-nanoparticle-mediated mitochondrial apoptosis augments adoptive T cell immunotherapy

**DOI:** 10.1016/j.xcrm.2026.102706

**Published:** 2026-03-30

**Authors:** Jiayan Fu, Yaoqi Liu, Zhenyu Zhong, Benyuan Cao, Luna Ran, Zijia Guo, Haiyang Dong, Nengcheng Bao, Rongqing Pan, Jinqiang Wang, Yuanhui Mao, Yongfeng Jin

**Affiliations:** 1 National Key Laboratory of Advanced Drug Delivery and Release Systems, Zhejiang University, Hangzhou, China; 2 MOE Laboratory of Biosystems Homeostasis & Protection, Innovation Center for Cell Signaling Network, College of Life Sciences, Zhejiang University, Hangzhou, China; 3 Institute of Immunology, Bone Marrow Transplantation Center of The First Affiliated Hospital & Liangzhu Laboratory, Zhejiang University, Hangzhou, China; 4 Department of Urology of The Second Affiliated Hospital & Liangzhu Laboratory, Zhejiang University, Hangzhou, China; 5 Emergency and Trauma Centre, First Affiliated Hospital, School of Medicine, Zhejiang University, Hangzhou, China; 6 School of Life Science, Zhejiang Chinese Medical University, Hangzhou, China

**Keywords:** adoptive T cell therapy, therapeutic mRNA, mitochondrial apoptosis, solid tumor, synergistic effect

## Abstract

Adoptive T cell therapy (ACT) holds promise for cancer immunotherapy, yet its clinical efficacy against solid tumors remains suboptimal. An emerging strategy aims to enhance ACT by modulating mitochondrial apoptosis (mtApoptosis) priming of cancer cells. This study develops an mRNA-based combinational strategy that utilizes mRNA lipid nanoparticles encoding BH3 domains from activator-type proteins to trigger robust mtApoptosis, thereby augmenting antitumor immunity with ACT. This approach preferentially induces immunogenic cell death in cancer cells and remodels the immunosuppressive microenvironment. Combined with ACT, the formulation synergistically enhances tumor cell killing *in vitro* by lowering the apoptotic threshold. *In vivo*, the combination improves therapeutic efficacy by boosting endogenous T cell cytotoxicity and mitigating ACT-induced T cell dysfunction. Single-cell transcriptomics further reveals that the combination reprograms effector T cells toward memory-like states with expanded TCR diversity. Collectively, this study proposes a combinatorial mRNA-based strategy and provides mechanistic insights for augmenting ACT through mtApoptosis priming.

## Highlights

• mRNA encoding BH3 activators triggers mtApoptosis and enhances antitumor immunity
• The combination synergistically enhances T-cell-mediated killing
• The combination promoting trafficking and polyfunctionality of effector T cells
• Transcriptomics reveals reprogramming of T cells toward memory-like states

## Graphical abstract

**Figure:**
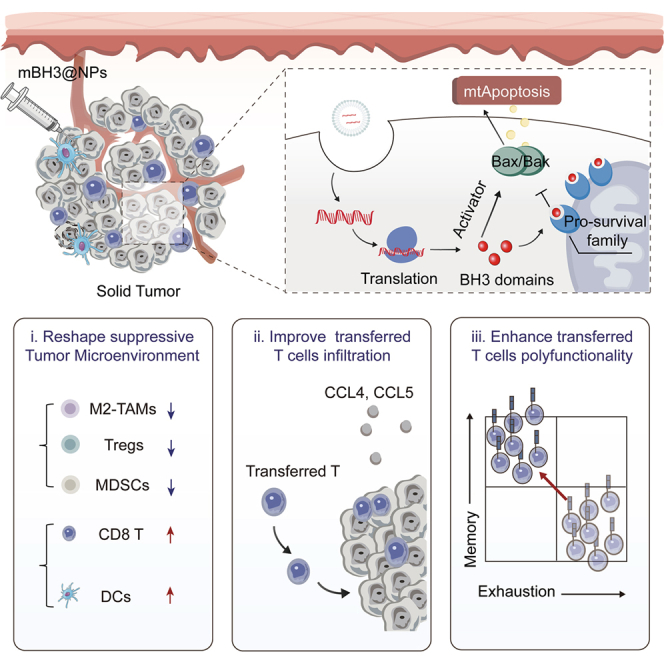

## Teaser

Fu et al. develop an mRNA/LNP strategy that primes cancer cells for mitochondrial apoptosis. This approach synergizes with adoptive T cell therapy to overcome immunosuppression, reinvigorate cytotoxic T cell responses, and foster memory-like states, thereby enhancing antitumor immunity against solid tumors.

## Introduction

Adoptive T cell therapy (ACT) has emerged as a highly promising cancer immunotherapy over the past decades and has achieved clinical success in the treatment of hematologic malignancies. However, its clinical efficacy against solid tumors remains limited,^1^^,^^2^^,^^3^ largely due to the immunosuppressive tumor microenvironment and intrinsic resistance of cancer cells, both of which hinder T-cell-mediated cytotoxicity and lead to T cell dysfunction.^4^^,^^5^^,^^6^^,^^7^ A promising strategy to enhance ACT efficacy involves sensitizing cancer cells by modulating their intrinsic mitochondrial apoptosis (mtApoptosis) priming, thereby synergizing with cytotoxic T cells by engaging non-overlapping mechanisms.^8^^,^^9^^,^^10^^,^^11^

The mtApoptosis pathway is regulated by interactions among three groups of BCL-2 family proteins: pro-survival proteins (e.g., BCL-2, BCL-X_L_, and MCL-1), pro-apoptotic BH3-only proteins further subdivided into activators (e.g., BIM, PUMA, and tBID) and sensitizers (e.g., BAD and NOXA), and the death effector proteins (BAX and BAK).^12^^,^^13^ During mtApoptosis, BAK/BAX activation represents a critical step that is tightly regulated by the balance between pro-survival and pro-apoptotic signals. Upon activation, BAK/BAX undergoes conformational oligomerization, leading to irreversible mitochondrial outer membrane permeabilization (MOMP) and subsequent initiation of downstream apoptotic cascades that dismantle cells.^14^^,^^15^^,^^16^ Current mtApoptosis-based therapeutics, including BH3 mimetics and BH3-derived peptides, are designed to selectively antagonize specific pro-survival BCL-2 family members.^17^^,^^18^^,^^19^^,^^20^^,^^21^ However, most such therapies have shown suboptimal efficacy as single agents against solid tumors due to the functional redundancy among multiple pro-survival BCL-2 members,^22^^,^^23^ rendering single-target antagonization insufficient.^24^ More importantly, merely releasing the inhibitory “brake” imposed by pro-survival BCL-2 members without providing sufficient direct pro-apoptotic “acceleration” fails to trigger effective apoptosis cascades. These limitations underscore the urgent need to develop potent mtApoptosis-based strategies to augment antitumor immunity with ACT.

mRNA-based therapeutics have emerged as potent alternatives to small molecules and peptides, enabling transient yet sustained protein expression. Moreover, mRNA possesses intrinsic immunostimulatory properties, acting as a self-adjuvant to reshape the tumor microenvironment.^25^^,^^26^^,^^27^^,^^28^^,^^29^^,^^30^^,^^31^ Building upon this, we sought to leverage mRNA encoding functional domains derived from pro-apoptotic proteins to overcome the aforementioned resistance. Specifically, these domains are designed not only to relieve the inhibitory constraints imposed by redundant pro-survival BCL-2 family members^32^^,^^33^ but also to directly activate death effectors, thereby priming robust mtApoptosis cascades and effective antitumor immune responses.^34^^,^^35^^,^^36^

Herein, this study developed an mRNA-based combinatorial strategy that employs mRNA lipid nanoparticles (LNPs) encoding BH3 domains from activator-type proteins to trigger robust mtApoptosis, thereby augmenting antitumor immunity with ACT. The mRNA/LNP formulation preferentially induces immunogenic cell death (ICD) in cancer cells and reshapes the immunosuppressive tumor microenvironment. Remarkably, its combination with ACT synergistically enhances cancer cell killing *in vitro* by lowering their apoptotic threshold. This synergistic effect was confirmed *in vivo*, where the combination therapy further revealed a pronounced synergistic effect in enhancing therapeutic efficacy by promoting the trafficking and polyfunctionality of infiltrating effector T cells. This enhanced immune response was achieved through a functional division of labor, simultaneously boosting endogenous T cell cytotoxicity and alleviating transferred T cell dysfunction. Single-cell analysis further revealed that this combination therapy reprogrammed effector T cells toward a more memory-like transcriptional profile with expanded TCR diversity. Collectively, this study not only implements an mRNA-based combinatorial strategy but also provides mechanistic insights into augmenting T cell killing through mtApoptosis priming.

## Results

### Design and engineering of mRNA/LNPs encoding distinct BH3 domains

**Figure 1:**
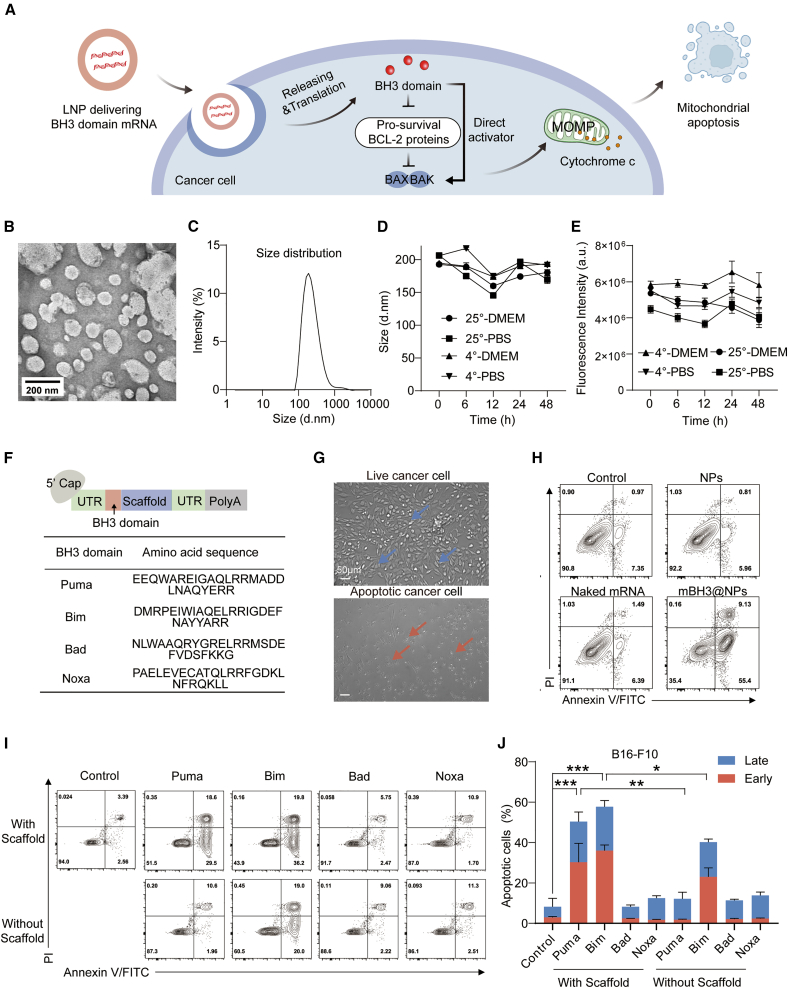
Design and engineering of mRNA/LNP encoding distinct BH3 domains (A) Schematic illustration of delivering mBH3@NPs to induce mtApoptosis in cancer cells. (B) Representative morphology of mBH3@NPs imaged by transmission electron microscope (TEM). Scale bars, 200 nm. (C) Size distribution of mBH3@NPs measured by dynamic light scattering (DLS). (D) Stability of mBH3@NPs in culture medium or PBS over 48 h at 25°C or 4°C, as measured by particle size changes. (E) Functional integrity of mNluc@NPs in culture medium or PBS over 48 h at 25°C or 4°C, as quantified by Nluc intensity (a.u.). (F) Schematic of mRNA constructs encoding scaffold-anchored BH3 domains derived from distinct pro-apoptotic BH3-only proteins (PUMA, BIM, BAD, and NOXA). (G) Transfection with mBH3@NPs for 8 h induced membrane blebbing and apoptotic bodies formation in CT-26 cancer cells. Scale bars, 50 μm. (H) Flow cytometric analysis of apoptotic B16-F10 cancer cells after treatment with mBH3@NPs (mBim@NPs shown as an example) for 8 h. (I and J) Flow cytometric analysis (I) and quantification (J) of apoptotic B16-F10 cancer cells after treatment with mRNA/LNP formulations encoding distinct BH3 domains, with or without scaffold for 8 h (*n* = 3). Two-tailed unpaired Student’s *t* test. All data are presented as the mean ± SD. ∗*p* < 0.05; ∗∗*p* < 0.01; ∗∗∗*p* < 0.001; NS, not significant. See also Figure S1 and Table S1.

To induce mtApoptosis, mRNA constructs were designed to encode functional domains from two distinct subgroups of BH3-only proteins: direct activators (Bim and Puma) and sensitizers (Bad and Noxa). This design strategy avoids the broad cytotoxicity associated with full-length proteins.^37^ These mRNAs were synthesized via *in vitro* transcription with ARCA capping and encapsulated into LNPs composed of the ionizable lipid SM-102, cholesterol CHO-HP, DSPC, and DMG-PEG2000,^38^ collectively termed mBH3@NPs (Figure 1A). This widely utilized LNP system is renowned for its high translation potential, providing a robust delivery platform for our mRNA constructs. mBH3@NPs exhibited uniform morphology and hydrodynamic size distribution, as confirmed by transmission electron microscope (TEM) and dynamic light scattering (DLS) (Figures 1B and 1C), and maintained physical and functional stability under various pathological conditions (Figures 1D and 1E).

Each BH3 domain was fused to a Stefin A quadruple aptamer scaffold to stabilize and spatially constrain its conformation, thereby achieving optimal interaction with pro-survival BCL-2 family proteins^39^^,^^40^ (Figure 1F). Agarose gel assay confirmed mRNA size and integrity with or without the scaffold (Figure S1A). In melanoma cells, mBH3@NPs (particularly mPuma@NPs and mBim@NPs) induced dose-dependent mtApoptosis, evidenced by apoptotic bodies, cell blebbing, and annexin V/PI staining (Figures 1G, 1H, and S1B). Among the four mBH3@NPs, BH3 domains from direct activators (PUMA and BIM) showed superior pro-apoptotic activity compared to sensitizers (Noxa and Bad), likely due to their broad affinity for pro-survival BCL-2 proteins and direct activation ability independent of C-terminal targeting domains^41^^,^^42^ (Figures 1I and 1J). Scaffold fusion further enhanced pro-apoptotic activity, particularly for Puma, consistent in colon cancer cell lines (Figures 1I, 1J, and S1C). Based on these results, this study selected scaffold-engineered BH3 domains derived from the activator subgroup (Bim andPuma)^43^^,^^44^ for further investigation in solid tumor immunotherapy.

### mBH3@NPs preferentially induced mtApoptosis and immunogenic cell death in cancer cells

**Figure 2:**
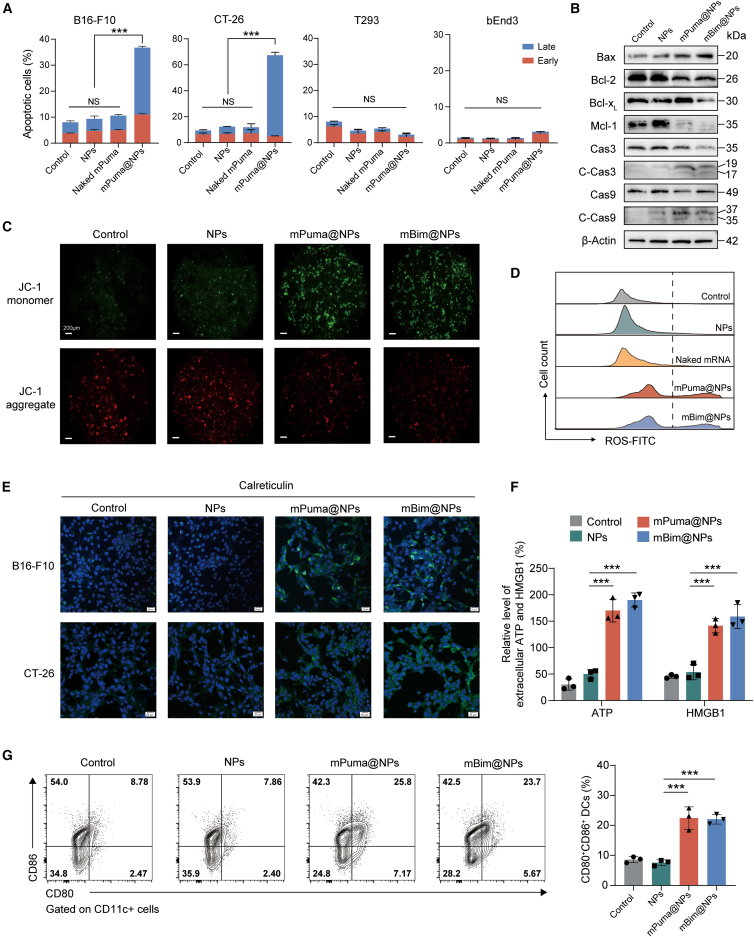
mBH3@NPs preferentially induced mtApoptosis and immunogenic cell death in cancer cells (A) Flow cytometry analysis of cells positive for Annexin V and propidium iodide (PI) in cancer and non-cancer cell lines after treatment with PBS (control), NPs, naked Puma mRNA, and mPuma@NPs for 12 h (*n* = 3). (B) Western blot analysis of mitochondrial apoptois pathway in CT-26 cells treated with control, NPs, mPuma@NPs, and mBim@NPs. Bax, Bcl-2, Bcl-x_L_, Mcl-1, caspase-3, cleaved caspase-3 (C-Cas3), caspase9, and cleaved caspase-9 (C-Cas9) proteins were detected. β-Actin was used as the loading control (*n* = 3). (C) Confocal laser scanning microscopy (CLSM) images of the 5,5′,6,6′-tetrachloro-1,1′,3,3′-tetraethyl-imidacarbocyanine (JC-1) probe in CT-26 cells after treatment with PBS (control), NPs, mPuma@NPs, and mBim@NPs for 12 h. Increased JC-1 monomer signal (green) and decreased JC-1 aggregate signal (red) indicate a decrease in mitochondrial membrane potential (*n* = 3). Scale bars, 200 μm. (D) Flow cytometry analysis of cellular oxygen species (ROS) levels using 2,7-dichlorofluorescein diacetate (DCFH-DA) staining in CT-26 cells after 12 h incubation with PBS (control), NPs, naked mRNA, mPuma@NPs, and mBim@NPs (*n* = 3). (E) CLSM images of CRT expression in B16-F10 and CT-26 cells after 12 h incubation with PBS (control), NPs, mPuma@NPs, and mBim@NPs (*n* = 3). Scale bars, 20 μm. (F) Extracellular ATP and HMGB1 expression levels were analyzed by ELISA in B16-F10 cells after 12-h incubation with PBS (control), NPs, mPuma@NPs, and mBim@NPs (*n* = 3). (G) Flow cytometry analysis and quantification of immune stimulation in BMDCs co-cultured with B16-F10 cells pretreated with PBS (control), NPs, mPuma@NPs, and mBim@NPs for 12 h, followed by 48-h co-culture (*n* = 3). One-way ANOVA with Tukey’s multiple comparisons test was used for all statistical analyses. Data are presented as the mean ± SD. ∗*p* < 0.05; ∗∗*p* < 0.01; ∗∗∗*p* < 0.001; NS, not significant. See also Figure S2.

The pro-apoptotic efficacy of mBH3@NPs was evaluated in both cancer and non-cancer cell lines, as theoretically, cancer cells expressing high levels of the pro-survival BCL-2 proteins are more susceptible to BH3-mediated apoptotic stimuli.^45^ Notably, both mBH3@NPs (mPuma@NPs and mBim@NPs) effectively induced apoptosis in murine melanoma (B16-F10) and colorectal carcinoma (CT-26) cell lines, while exhibiting minimal toxicity toward non-cancer cell lines (T293 and bEnd3) (Figures 2A, S2A, and S2B), thereby supporting the potential of BH3-domain-mediated mRNA therapy as a cancer immunotherapy with a favorable safety profile.^46^ To further elucidate the potential mechanisms underlying the antitumor effects of mBH3@NPs *in vitro*, key markers of the mtApoptosis pathway were evaluated via western blotting and immunofluorescence staining. The elevation in the Bax/Bcl-2 ratio following mBH3@NPs transfection reflects a shift toward apoptosis, thereby initiating the downstream cleavage cascade of effector caspases, including caspase-3 and caspase-9 (Figure 2B). The observed marked downregulation of Mcl-1 likely resulted from ubiquitin-mediated proteasomal degradation, serving as a secondary effect of apoptosis activation^47^ (Figure 2B). Notably, these phenomena were more pronounced with mBim@NPs, indicating their superior potency as apoptosis activators. Furthermore, both mBH3@NPs induced loss of mitochondrial outer membrane potential in cancer cells, as demonstrated by JC-1 staining (Figure 2C), and rapidly promoted reactive oxygen species (ROS) generation, triggering cytochrome *c* release from mitochondria—hallmark events required for initiating mtApoptosis (Figures 2D and S2C). Collectively, these results indicated that mBH3@NPs preferentially triggered mtApoptosis in cancer cell *in vitro*.

To investigate whether mBH3@NPs induce ICD via damage-associated molecular pattern (DAMP) release, key ICD biomarkers were detected, including surface calreticulin (CRT), extracellular HMGB1, and secreted ATP.^48^ Notably, mBH3@NPs markedly upregulated CRT exposure (an “eat me” signal; Figure 2E), along with the release of HMGB1 and ATP—representing “danger” and “find me” signals, respectively (Figures 2F and S2D). Co-culture of bone-marrow-derived dendritic cells (BMDCs) with mBH3@NP-pretreated cancer cells enhanced immune stimulation of DCs, manifested as elevated expression of costimulatory markers CD80 and CD86 (Figure 2G), indicating that mBH3@NPs-mediated DAMPs release promoted dendritic cell maturation. Collectively, mBH3@NPs preferentially induced both mtApoptosis and ICD in cancer cells.

### mBH3@NPs induced effective antitumor immunity and remodeled the tumor microenvironment

**Figure 3:**
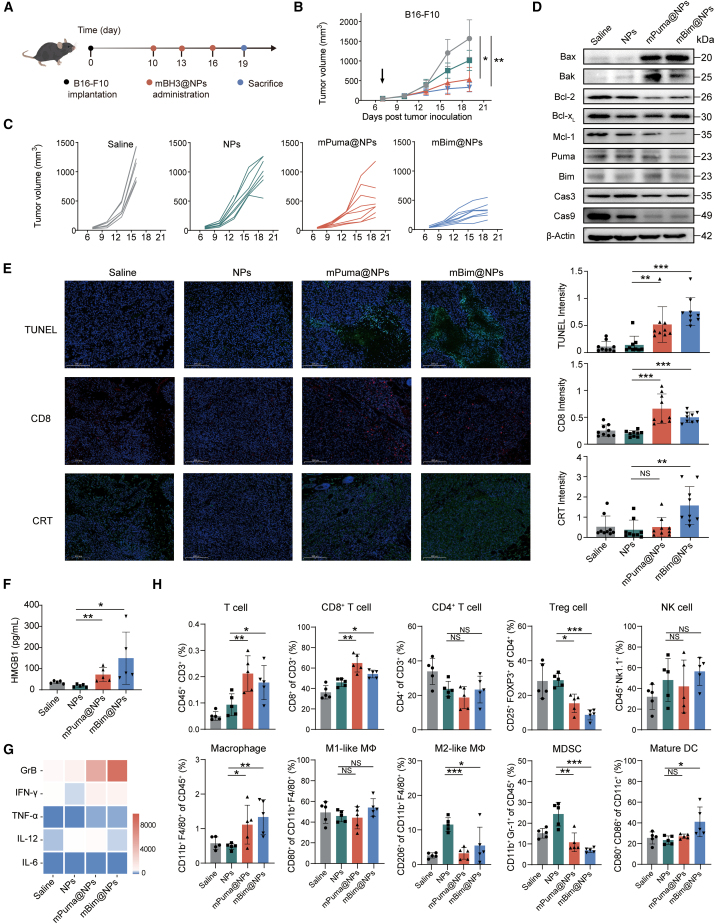
mBH3@NPs induced effective antitumor immunity and remodeled the tumor microenvironment (A) Experimental timeline for mBH3@NPs administration in B16-F10-tumor-bearing mice. Mice were administered with mBH3@NPs three times, with a dosing interval of every 2 days (B) Average tumor volume curves for mice treated in the melanoma model (*n* = 8). (C) Individual tumor volume curves for mice treated in the melanoma model (*n* = 8). (D) Western blot analysis of Bax, Bak, Bcl-2, Bcl-x_L_, Mcl-1, Puma, Bim, caspase-3, and caspase-9 expression following different treatments. β-Actin was used as a loading control (*n* = 3). (E) Representative images of tumor immunostaining and quantification for TUNEL (green)/DAPI (blue), CD8 (red)/DAPI (blue), and CRT (green)/DAPI (blue) at day 19 following the indicated treatments in the melanoma model. Scale bars, 200 μm. (F) HMGB1 expression in tumor tissues analyzed by ELISA following different treatments (*n* = 5). (G) Heatmap of cytokine expression levels (GrB, IFN-γ, TNF-α, IL-12, and IL-6) in tumor tissues following different treatments (*n* = 5). (H) Flow cytometric analysis of immune cell populations (T cells, CD8^+^ T cells, CD4^+^ T cells, Treg cells, NK cells, macrophages, M1-like TAMs, M2-like TAMs, and MDSCs) within tumors and CD11c^+^CD80^+^CD86^+^ mature dendritic cells in lymph nodes (*n* = 5). One-way ANOVA with Tukey’s multiple comparisons test was used for all statistical analyses. Data are presented as the mean ± SD. ∗*p* < 0.05; ∗∗*p* < 0.01; ∗∗∗*p* < 0.001; NS, not significant. See also Figures S3 and S4.

To investigate the *in vivo* therapeutic efficacy of mBH3@NPs, they were intratumorally administered into a subcutaneous melanoma model (Figure 3A). Complementary biodistribution studies performed using Nluc-encoding reporter mRNA further demonstrated that the formulation was predominantly retained within the tumor tissue (Figures S3A and S3B). Both mBim@NPs and mPuma@NPs suppressed tumor growth, with mBim@NPs exhibiting superior therapeutic effects (Figures 3B, 3C, and S4A), consistent with their pro-apoptotic activity observed *in vitro*. Mechanistically, mBH3@NPs-treated tumors exhibited a notable increase in Bax/Bcl-2 and Bak/Bcl-2 ratios, alongside reduced levels of pro-caspase-3 and pro-caspase-9 (Figure 3D), confirming robust apoptosis activation. Meanwhile, endogenous expression levels of corresponding BH3-only proteins (PUMA and BIM) remained largely unchanged after transfection (Figure 3D). TUNEL staining further validated the activation of apoptosis (Figure 3E, upper panel). Additionally, immunofluorescence showed elevated CD8^+^ T cell infiltration in both mBH3@NPs-treated groups compared to the controls (Figure 3E, middle panel). Notably, mBim@NPs treatment induced stronger CRT exposure and HMGB1 release (Figures 3E, lower panel, and 3F), indicating a more potent induction of ICD *in vivo*.

To better understand their immunomodulatory effects, tumor inflammatory cytokines and immune cell profiles were analyzed. Both mPuma@NPs and mBim@NPs significantly elevated secreted levels of granzyme B (GrB), interferon gamma (IFN-γ), and tumor necrosis factor alpha (TNF-α) within tumors (Figure 3G). Immune profiling revealed a marked enrichment of tumor-infiltrating T cells (particularly CD8^+^ cytotoxic T cells) in mBH3@NPs-treated tumors, accompanied by a concurrent reduction in immunosuppressive cell populations, including inhibitory regulatory T cells (Tregs), myeloid-derived suppressor cells (MDSCs), and M2-like tumor-associated macrophages (M2-TAMs) (Figure 3H). These shifts likely resulted from large-scale ICD-driven immune activation and downregulation of key pro-survival proteins critical for suppressive cell survival.^49^^,^^50^ Notably, the immunomodulatory effects were more pronounced in the mBim@NPs-treated group, indicating its stronger capacity to alleviate the immunosuppressive TME. Moreover, mBim@NPs markedly enhanced dendritic cell maturation in tumor-draining lymph nodes (TdLNs) (Figure 3H), suggesting augmented systemic immune priming. Importantly, no significant body weight loss, hepatotoxicity, or organ damage was observed (Figures S3C–S3F), indicating favorable systemic biosafety. We further evaluated the efficacy of mBH3@NPs in the MC-38 tumor model (Figures S4A–S4C). Compared to the melanoma model, they elicited moderate tumor growth inhibition. Ki67 and TUNEL immunohistochemistry staining confirmed decreased cell proliferation and increased apoptosis in both mBH3@NPs-treated tumors (Figures S4D and S4E). These observations suggest that mBH3@NPs alone can delay tumor progression but may be insufficient to control aggressive tumor progression.

Overall, mBH3@NPs enhanced the exposure of ICD signals and promoted dendritic cell maturation and cross-priming of effector T cells, thereby reshaping the immunosuppressive TME and initiating antitumor immunity cycles.

### mBH3@NPs synergized T-cell-mediated cytotoxicity *in vitro*

**Figure 4:**
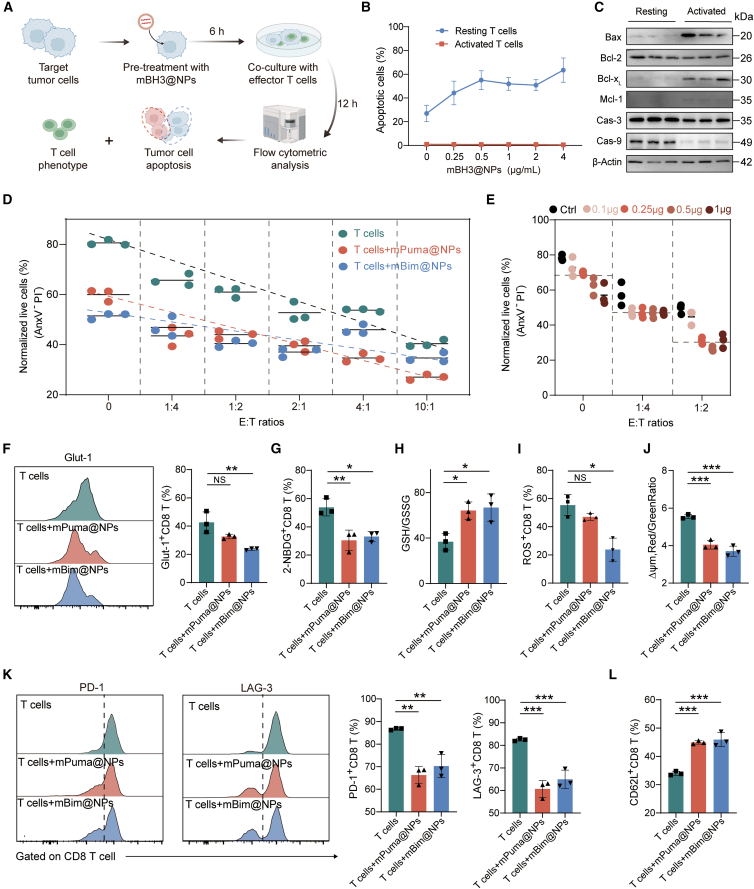
mBH3@NPs synergized T-cell-mediated cytotoxicity *in vitro* (A) Workflow of procedures for evaluating T-cell-mediated killing effects in target cancer cells. (B) Sensitivity of freshly isolated resting T cells and activated T cells to mBH3@NPs treatment (*n* = 3). (C) Western blot analysis of altered protein expression in T cells after pre-activation for 3 days. Bax, Bcl-2, Bcl-x_L_, Mcl-1, caspase-3, and caspase-9 were detected. β-Actin was used as a loading control (*n* = 3). (D) Quantification of B16-OVA tumor cell sensitivity to OT-1 T-cell-mediated killing following treatments of PBS, mPuma@NPs, and mBim@NPs at different E:T ratios (1:4, 1:2, 2:1, 4:1, and 10:1) by flow cytometry (*n* = 3). (E) Flow cytometric quantification of B16-OVA tumor cell sensitivity to OT-1 T-cell-mediated killing following different dosages of mPuma@NPs (0.1, 0.25, 0.5, and 1 μg) at lower E:T ratios (*n* = 3). (F) Flow cytometry histograms and quantification of Glut-1 OT-1 T cells co-cultured with B16-OVA cells treated with PBS, mPuma@NPs, and mBim@NPs. The co-culture was performed at an E:T ratio of 1:1 (*n* = 3). (G) Glucose uptake capacity in OT-1 T cells was measured by flow cytometry using the 2-NBDG assay. (H) The cellular GSH:GSSG ratio was measured in OT-1 T cells after a 12-h co-culture with B16-OVA cells pre-treated with PBS, mPuma@NPs, and mBim@NPs. (I) Flow cytometry analysis of ROS levels of OT-1 T cells co-cultured with B16-OVA cells treated with PBS, mPuma@NPs, and mBim@NPs (*n* = 3). (J) Mitochondrial membrane potential (Δψm) in OT-1 T cells was assessed by flow cytometry using the JC-1 probe after co-culture with B16-OVA cells treated with PBS, mPuma@NPs, and mBim@NPs. The Δψm is presented as the ratio of red (aggregates, high Δψm) to green (monomers, low Δψm) fluorescence intensity (*n* = 3). (K) Flow cytometry histograms and quantification of PD-1 and LAG-3 expression of OT-I T cells co-cultured with B16-OVA cells treated with PBS, mPuma@NPs, and mBim@NPs (*n* = 3). (L) Flow cytometry analysis of frequency of CD62L of OT-1 T cells co-cultured with B16-OVA cells treated with PBS, mPuma@NPs, and mBim@NPs (*n* = 3). One-way ANOVA with Tukey’s multiple comparisons test was used for all statistical analyses. Data are presented as the mean ± SD. ∗*p* < 0.05; ∗∗*p* < 0.01; ∗∗∗*p* < 0.001; NS, not significant. See also Figure S5.

Given the potential of mtApoptosis-based therapeutics to augment immunotherapies,^8^^,^^9^^,^^10^ we evaluated whether the developed BH3-domain-mediated mRNA therapy could synergize with cytotoxic T cells *in vitro*. Co-culture assays were performed using B16-OVA target cells and OT-1-derived effector cells (Figure 4A). CD3/CD28-pre-activated cytotoxic T lymphocytes (CTLs) exhibited resistance to mBH3@NPs-induced apoptosis (Figure 4B), likely due to an elevated mtApoptosis threshold driven by upregulation of both pro-survival and pro-apoptotic proteins (Figure 4C). This intrinsic resistance in pre-activated CTLs provided a rationale for the safety of combining mBH3@NPs with T cell therapy.

Next, the synergistic effects of two mBH3@NPs (mPuma@NPs and mBim@NPs) on T-cell-mediated cytotoxicity were evaluated at varying effector-to-target (E:T) ratios. Both formulations enhanced T-cell-mediated killing, rendering target cells more susceptible to CTL attack (Figure 4D). Surprisingly, the comparatively moderate apoptotic induction by mPuma@NPs allowed for a greater enhancement of T-cell-mediated killing, resulting in a more pronounced synergistic effect at increased E:T ratios (Figure 4D). In contrast, the stronger apoptosis induction by mBim@NPs may have led to near-saturation of the synergistic effect. Furthermore, synergy was most evident at lower E:T ratios (1:4 or 1:2) and diminished at higher ratios (4:1 or 10:1), consistent with previous findings in natural killer (NK)-cell-based therapy.^9^ These results suggest that T-cell-mediated cytotoxicity relies more heavily on intrinsic apoptotic priming at lower E:T ratios, whereas cytotoxic function tends toward saturation at higher ratios. Notably, this combination therapy achieves comparable killing efficacy to that of T cells alone at high ratios (10:1) even at lower E:T ratios, suggesting its potential to shift the E:T balance in favor of T cells and reduce the cell dose required for clinical applications. Given that lower E:T ratios better mimic the immunosuppressive conditions of the solid tumor microenvironment,^51^ the synergistic effects of varying mBH3@NPs doses were examined at low E:T ratios (1:4 and 1:2). Surprisingly, extremely low doses of mBH3@NPs were sufficient to augment T-cell-mediated cytotoxicity by pushing cancer cells beyond their apoptosis threshold, with higher doses yielding no additional benefit. This non-linear dose-response suggested that combination therapy could reduce mRNA requirements and associated costs (Figure 4E). Furthermore, our calculated combination index (CI) consistently remained below 1 (Figures S5A and S5B).

Within the nutrient-deprived and metabolically suppressive tumor microenvironment, T cells often undergo dysfunctional metabolic reprogramming, ultimately leading to exhaustion.^52^^,^^53^ To further elucidate the molecular mechanism underlying the synergy between mBH3@NPs and T cells, the alterations in T cell metabolic state and mitochondrial function were investigated. It was found that T cells co-cultured with mBH3@NPs-pre-treated tumor cells exhibited significantly reduced glucose uptake, as evidenced by downregulation of Glut-1 expression and decreased 2-NBDG incorporation (Figures 4F and 4G). Concurrently, these T cells displayed a marked alleviation of oxidative stress, characterized by an elevated GSH/GSSG ratio, reduced ROS levels, and decreased mitochondrial membrane potential (Figures 4H–4J). These metabolic alterations are closely coupled with a functional shift: the expression of exhaustion markers PD-1 and LAG-3 was significantly downregulated (Figure 4K), while the proportion of CD62L^+^ T cells with memory potential increased (Figure 4L). mBH3@NPs also enhanced the secretion of cytotoxic factors including GrB and IFN-γ (Figures S5C and S5D), confirming the improvement in T cell function (Figure S5E). Collectively, our data demonstrate that mBH3@NPs not only sensitize tumor cells to apoptosis but also remodel the hostile metabolic milieu of T cells, thereby enhancing their metabolic fitness, delaying exhaustion, and promoting memory differentiation.

### mBH3@NPs promoted the recruitment of adoptively transferred T cells

**Figure 5:**
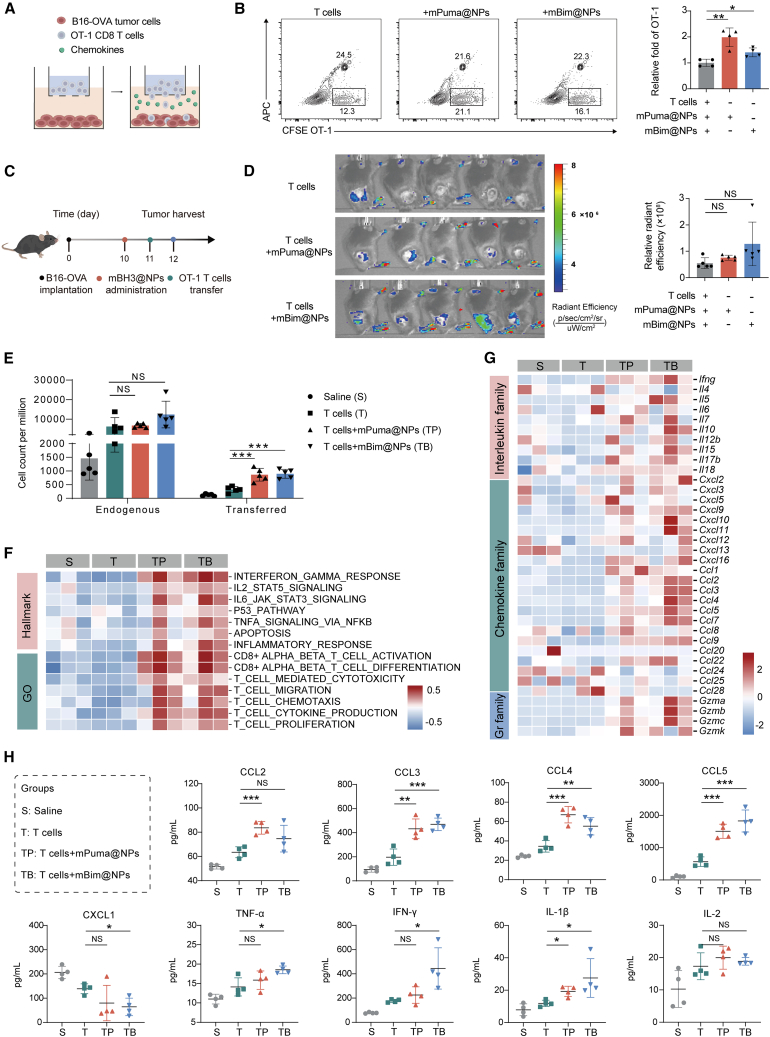
mBH3@NPs promoted the recruitment of adoptively transferred T cells (A) Schematic of transwell migration assay using B16-OVA cells and preactivated OT-1 T cells. (B) Transwell migration assay of OT-I T cells (CFDA-labeled). Representative flow cytometry graphs and quantification of T cell infiltration relative fold changes (calculated as the ratio of bottom well-located OT-I T cells to counting beads) (*n* = 3). (C) Experimental timeline for mBH3@NPs administration combined with adoptively transferred T cells in B16-OVA tumor-bearing mice. (D) Serial IVIS images and quantification of infiltrating Cy5-T cell signals in B16-OVA tumor-bearing mice (*n* = 5). (E) Quantification of tumor-infiltrating endogenous T cells and transferred CD8^+^ T cells per million cells (*n* = 5). (F) Heatmap of RNA-seq data for key interleukin family member, chemokine family member, and granzyme family member from harvested tumor tissues, plotted as *Z* score of normalized gene expression for each gene (*n* = 3). (G) Heatmap of the differences in pathway activities scored by GSVA from harvested tumor tissues, plotted as *Z* score of normalized gene expression for each gene (*n* = 3). (H) Validation of key cytokines and chemokines expression by multiplex cytokine assay. Tumor tissue was homogenized, and the cell supernatant of each treatment group was collected (*n* = 4). One-way ANOVA with Tukey’s multiple comparisons test was used for all statistical analyses. Data are presented as the mean ± SD. ∗*p* < 0.05; ∗∗*p* < 0.01; ∗∗∗*p* < 0.001; NS, not significant. See also Figure S6.

Inadequate T cell infiltration is a major barrier limiting the therapeutic efficacy of ACT in solid tumors.^54^ Given that mBH3@NPs monotherapy induced potent ICD and initiated antitumor immune cycle *in vivo*, this study investigated whether they could enhance the recruitment of adoptively transferred T cells. In a Transwell assay, fluids collected from mBH3@NPs-treated tumors significantly increased the migration of CFSE-labeled OT-1 T cells toward B16-OVA cells, with a 2-fold and 1.5-fold increase observed in the mPuma@NPs and mBim@NPs groups, respectively (Figures 5A and 5B). Building on these *in vitro* observations, we further assessed *in vivo* T cell infiltration by intravenously injecting Cy5-labeled, pre-activated OT-1 T cells one day after mBH3@NPs administration (Figure 5C). *In vivo* imaging showed increased tumor accumulation of Cy5-labeled T cells in mBH3@NPs-treated groups, though not statistically significant (Figure 5D). Flow cytometric analysis of excised tumors further confirmed a significant increase in OT-1 T cell counts in both the mPuma@NPs and mBim@NPs groups (Figure 5E).

To further elucidate the underlying mechanisms, transcriptomic analyses were performed. The heatmap revealed upregulation of pro-inflammatory interleukins (ILs), chemokines, and granzymes (key mediators of T cell recruitment and activation) in mBH3@NPs-treated groups (Figure 5F). Pathway enrichment analysis further supported these findings, highlighting enhanced immune-related signaling pathways, particularly those involved in T cell activation, migration, and proliferation (Figure 5G). Consistent with transcriptomic data, multiplex cytokine assays confirmed elevated protein levels of key pro-inflammatory chemokines (CCL2, CCL3, CCL4, and CCL5) and cytokines (TNF-α, IFN-γ, and IL-1β) in mBH3@NPs-treated tumors (Figure 5H). Among these, the CCL4/CCL5-CCR5 axis has been identified as a robust correlate of CD8^+^ T cell infiltration.^55^^,^^56^^,^^57^ Based on this axis, co-culture of mBH3@NPs-treated tumor cells with BMDCs led to elevated CCL4 and CCL5 secretion in both mPuma@NPs and mBim@NPs groups (Figures S6A and S6B). Furthermore, Transwell migration assays showed that supernatants from mBim@NPs-treated cells induced the highest CD8^+^ T cell migration (Figure S6C), underscoring the pivotal role of CCL4 and CCL5 in T cell trafficking. Taken together, these results suggest that mBH3@NPs enhance the recruitment of adoptively transferred T cells by stimulating the secretion of key chemokines and cytokines within the tumor microenvironment.

### The combination of mBH3@NPs and adoptive T cell therapy improved therapeutic effects

**Figure 6:**
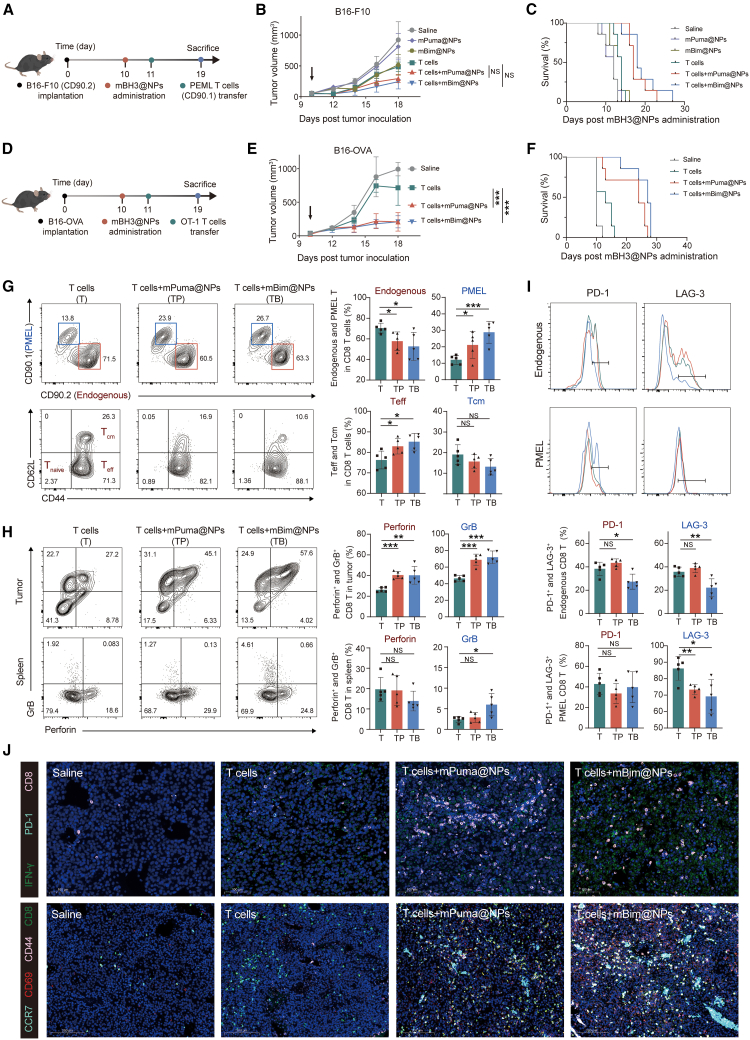
mBH3@NPs combined with adoptive T cell therapy improved the therapeutic effect (A) Experimental timeline for mBH3@NPs administration combined with adoptively transferred T cells from PMEL mice in B16-F10-tumor-bearing mice. (B) The average tumor volume curves for mice treated in the B16-F10-tumor-bearing mice model (*n* = 7). (C) Survival analysis for mice treated in B16-F10-tumor-bearing mice model (*n* = 7). Mice were humanely euthanized when tumor volume reached the predefined ethical endpoint (15 mm × 15 mm). The *x* axis indicates days post-mBH3@NPs administration. (D) Experimental timeline for mBH3@NPs administration combined with adoptive transfered T cells from OT-1 mice in B16-OVA-tumor-bearing mice. (E) The average tumor volume curves for mice treated in B16-OVA-tumor-bearing mice model (*n* = 7). (F) Survival analysis for mice treated in B16-OVA-tumor-bearing mice model (*n* = 7). Mice were humanely euthanized when tumor volume reached the predefined ethical endpoint (15 mm × 15 mm). (G) Representative flow cytometry plot and quantification of CD44 and CD62L expression levels gated on total CD8^+^ T cells (upper panel) and the percentage of endogenous (CD90.2) CD8^+^ T cells and transferred PMEL (CD90.1) CD8^+^ T cells gated on total CD8^+^ T cells (lower panel) (*n* = 5). (H) Representative flow cytometry plot and quantification of the expression level of granzyme B (GrB) and perforin in total CD8^+^ T cells from tumors and spleens (*n* = 5). (I) Representative flow cytometry plot and quantification of the expression level of PD-1 and LAG-3 in both endogenous and transferred PMEL CD8^+^ T cells from tumors (*n* = 5). (J) Immunofluorescence evaluation of CD8^+^ T cell exhaustion, effector, and memory function. Upper panel: representative immunofluorescence images of tumors from different groups for analyzing the exhaustion (PD-1^+^, cyan) and effector function (IFN-γ^+^, green) of CD8^+^ T cells (CD8^+^, pink). Scale bars, 100 μm. Lower panel: representative immunofluorescence images of tumor-infiltrating CD8^+^ T cells (CD8^+^, green) along with markers for activation (CD69^+^, red) and memory differentiation (CD44^+^, pink; CCR7^+^, cyan). Scale bars, 200 μm (*n* = 3). One-way ANOVA with Tukey’s multiple comparisons test was used for all statistical analyses. Data are presented as the mean ± SD. ∗*p* < 0.05; ∗∗*p* < 0.01; ∗∗∗*p* < 0.001; NS, not significant. See also Figures S7–S11.

We next evaluate the *in vivo* therapeutic effects of combining mBH3@NPs with ACT. The B16-F10 melanoma model expressing gp100 antigen was employed. When tumor volume reached ∼100 mm^3^, mBH3@NPs (mPuma@NPs and mBim@NPs) were administered intratumorally, followed by intravenous infusion of pre-activated gp100-specific PMEL T cells the next day (Figure 6A). This temporal separation was designed to minimize any potential adverse effects on the viability of infused T cells. While monotherapy with either mBH3@NPs or T cells moderately inhibited tumor growth, combination therapies showed markedly enhanced tumor suppression (Figures 6B, S7A, and S7B). Notably, the combination of mBim@NPs and T cells exhibited the strongest antitumor effect (Figure 6B). Survival analysis further confirmed the therapeutic benefit, with significantly prolonged survival in the combination groups (Figure 6C) and no notable changes in body weight (Figure S7C). These synergistic effects were also evident in the B16-OVA melanoma model using OT-1 T cells (Figure 6D), where combination therapy significantly suppressed tumor progression and improved survival compared to T cell monotherapy (Figures 6E, 6F, and S8A–S8C).

To elucidate the mechanisms underpinning the enhanced antitumor response, we analyzed the proportion, absolute quantity, phenotype, and function of tumor-infiltrating CD8^+^ T cells. Compared to ACT alone, co-treatment with mBH3@NPs markedly increased the absolute number of tumor-infiltrating effector T cells, including both endogenous and transferred PMEL populations, alongside elevated counts of T_eff_ and T_cm_ subsets, consistent with genuine expansion rather than a proportional shift (Figures S7D and S7E). A predominant increase was observed in the proportion of transferred PMEL T cells (CD90.1) (Figure 6G). Although combination therapy promoted a general shift of tumor-infiltrating CD8^+^ T cells from a central memory (CD44_high_CD62L_high_) to an effector memory (CD44_high_CD62L_low_) phenotype (Figure 6G), it appeared to foster a functional division of labor—enhancing the immediate cytotoxicity of endogenous cells while preserving long-term persistence by maintaining a central memory phenotype in transferred PMEL T cells, particularly when combined with mBim@NPs (Figure S7F). The combination therapy enhanced the polyfunctionality of tumor-infiltrating CD8^+^ T cells. Notably, this effect was most pronounced within the endogenous T cell compartment, where we observed a marked increase in the expression of key cytotoxic molecules, including GrB and perforin (Figures 6H and S7G). Moreover, the combination of T cells with mBim@NPs further boosted GrB secretion by splenic T cells upon *ex vivo* restimulation (Figure 6H), indicating enhanced systemic cytotoxicity. Beyond promoting cytotoxic function, the combination of T cells with mBim@NPs was also associated with modulated expression of key immune checkpoint molecules. Reduced levels of programmed cell death protein 1 (PD-1) and LAG-3 were observed on both endogenous and transferred CD8^+^ T cells (Figures 6I and S7H). Considering the analysis time points and the concomitant improvement in T cell effector function, this reduction may reflect an overall shift toward a more functional state. In contrast, the absence of modulated checkpoint profile observed in the combination of T cells with mPuma@NPs (Figures 6I and S7H) may correlate with the superior therapeutic outcome of the mBim@NPs combination, suggesting the establishment of a more favorable immunomodulatory landscape. To further validate the robustness of this combinatory strategy, similar experiments were conducted in the B16-OVA melanoma model. Similar to prior findings, the combination therapy of T cells with mBH3@NPs enhanced CD8^+^ T cell infiltration and polyfunctionality, including increased cytotoxicity and reduced dysfunction (Figures S8D–S8G). Immunofluorescence imaging further confirmed enhanced CD8^+^ T cell accumulation in tumors and lymph nodes in the combination groups, accompanied by a heightened effector state, manifested by increased IFN-γ expression and reduced inhibitory checkpoint PD-1 (Figures 6J and S9A–S9C). To specifically visualize and spatially resolve T cells with memory and tissue-adaptation potential, multiplex staining for CD8, CD44, CCR7, and CD69 was performed. This spatial analysis revealed that the combination therapy enriched tumor-infiltrating CD8^+^ T cells co-expressing CD44, CCR7, and CD69 (Figures 6J and S9A). The observed phenotype suggests that the combination therapy induced a differentiation state that blends memory-associated trafficking potential with tissue-adaptive features, potentially contributing to a more sustained immune response. No significant change was observed in liver and kidney function markers (ALT, AST, TBIL, and CR), with only a slight reduction in urea levels noticed in the combination of T cells and mBim@NPs group (Figure S8H). Furthermore, systematic monitoring of serum cytokines related to cytokine release syndrome (IL-6, IL-10, TNF-α, and IFN-γ) revealed no significant elevations, indicating a low risk of immune-related adverse events (Figure S8I). Overall, these findings underscore the enhanced potentiation of T cell polyfunctionality by the combination of ACT with mBH3@NPs, especially with mBim@NPs.

To further evaluate the performance of our platform, a head-to-head comparison was conducted against the clinically used BH3 mimetic ABT-199 (Venetoclax) in combination with adoptive T cell therapy. Results showed that both ABT-199 monotherapy and its combination with T cells showed moderate efficacy, whereas mBH3@NPs (mPuma@NPs or mBim@NPs) combined with T cells significantly inhibited tumor growth, prolonged survival, and enhanced T cell infiltration (Figures S10A–S10E). This direct comparison highlights the superior potential of the mRNA-LNP platform for enabling effective immunotherapy in solid malignancies.

To further evaluate the translational potential, a humanized xenograft model was established using HeLa-NY-ESO-1 cells (Figures S11A and S11B). In an *ex vivo* co-culture assay, pretreatment of tumor cells with both mPuma@NPs and mBim@NPs enhanced their susceptibility to TCR-T-cell-mediated killing. This synergistic effect was evidenced by increased apoptosis observed across both low and high E:T ratio (Figure S11C). Consistent with this, in a subcutaneous HeLa-NY-ESO-1 xenograft model in NSG mice, the combination of mBH3@NPs with TCR-T cells potently enhanced antitumor efficacy compared to T cells alone (Figures S11D–S11G). Collectively, these results demonstrated that mBH3@NPs synergized with antigen-specific T cells enhance tumor cell elimination in both human-cell-based assays and *in vivo* models.

### Combination therapy drove CD8^+^ T cells toward memory-like states with expanded TCR diversity

**Figure 7:**
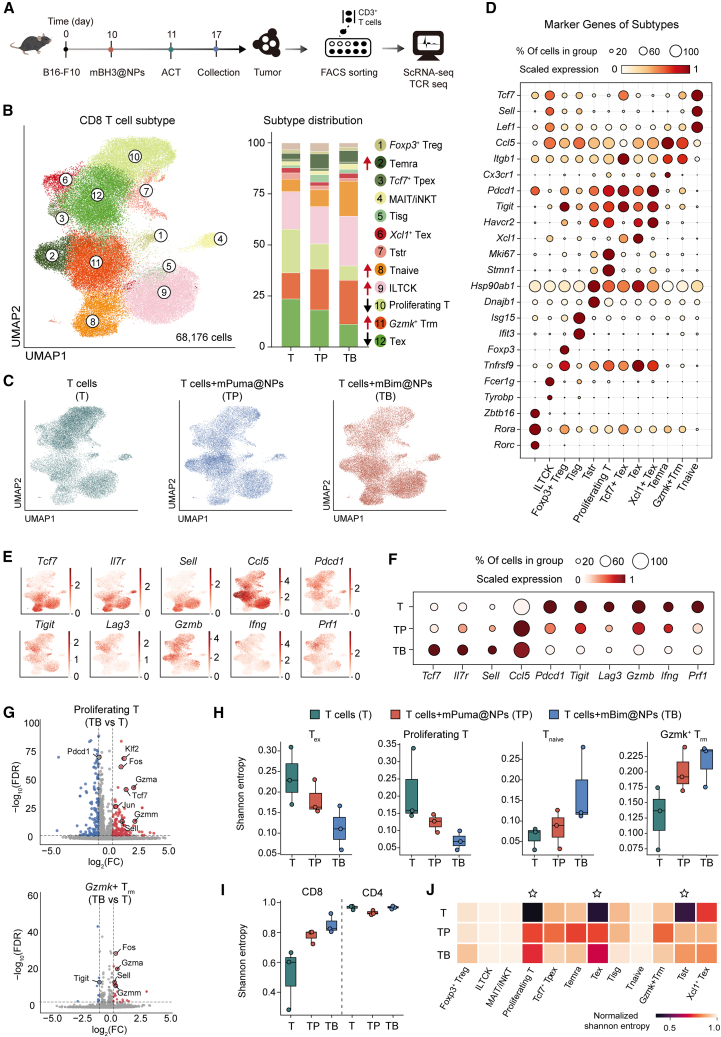
Combination therapy drove CD8^+^ T cells toward memory-like states with expanded TCR diversity (A) Experimental design for scRNA-seq. Mice were treated with ACT alone or combined with mBim@NPs and mPuma@NPs as shown (*n* = 3); CD3^+^ T cell isolated from mice for scRNA-seq. (B) UMAP representation and distribution of 68,176 cells of the CD8^+^ T cell atlas in each experimental condition, colored by 12 CD8^+^ T cell subtypes annotated in this study. (C) UMAP representation of the CD8^+^ T cells atlas colored by each experimental condition. (D) A dot plot showing the expression levels of marker genes in each CD8^+^ T cell subtype. The color of the dots indicates the average scaled expression level, and the size of the dots indicates the percentage of cells expressing the gene in each subtype. (E) Indicated expression of gene marker associated with memory (*Tcf7*, *Il7r*, *Sell*, and *Ccl5*), exhaustion (*Pdcd1*, *Tigit*, and *Lag3*), and cytotoxicity (*Gzmb*, *Ifng*, and *Prf1*) in the UMAP plot. (F) A dot plot showing the expression levels of marker genes in Figure 7E. The color of the dots indicates the average scaled expression level, and the size of the dots indicates the percentage of cells expressing the gene in each subtype. (G) Volcano plots of log_2_ (fold change) and log_10_ (adjusted FDR value) of differentially expressed genes in proliferating T and *Gzmk*+ T_rm_ from mice treated with ACT monotherapy and combination therapy with mBim@NPs. (H) Shannon entropy of the T_ex_, proliferating T, T_naive_, and *Gzmk*^+^ T_rm_ subsets across different treatment groups (T: ACT monotherapy; TP: ACT + mPuma@NPs; TB: ACT + mBim@NPs). (I) Shannon entropy of total CD8^+^ T cells and CD4^+^ T cells across different treatment groups. (J) A heatmap of normalized Shannon entropy for CD8^+^ T cell subsets across different treatment groups. See also Figure S12.

The aforementioned results demonstrated distinct polyfunctional states of tumor-infiltrating CD8^+^ T cells between ACT monotherapy and combination therapy with mBH3@NPs. To elucidate the transcriptional landscape disparities, we performed single-cell RNA sequencing (scRNA-seq) on these T cells (Figure 7A). Integrated and unsupervised clustering of 68,176 CD8^+^ T cells from three conditions resolved 12 transcriptionally distinct clusters: *Foxp3*^+^ regulatory T cells (cluster 1), terminally differentiated effector memory or effector cells (T_emra_, cluster 2), *Tcf*^+^ precursor exhausted T cells (*Tcf*^+^ T_pex_, cluster 3), mucosal-associated invariant/invariant NK T cells (MAIT/iNKT, cluster 4), interferon-stimulated gene-positive T cells (T_isg_, cluster 5), *Xcl1*^+^ exhausted T cells (*Xcl1*^+^ T_ex_, cluster 6), stress response T cells (T_str_, cluster 7), naive T (T_naive_, cluster 8), *Fcer1g*^+^ innate-like T cells (ILTCK, cluster 9), proliferating T (cluster 10), *Gzmk*^+^ tissue-resident memory cells (*Gzmk*^+^ T_rm_, cluster 11), and terminally exhausted T cells (T_ex_, cluster 12) (Figures 7B–7D).^58^^,^^59^^,^^60^

Analysis of tumor-infiltrating lymphocyte (TIL) phenotypes revealed that the mBim@NPs-based combination induced a more pronounced reorganization of the CD8^+^ TILs landscape compared to the mPuma@NPs group (Figure 7B). Distinct CD8^+^ T cell subset distributions were observed across treatment groups. ACT monotherapy responders were dominated by T_ex_ and proliferating T, with the latter cluster co-expressing high levels of mitotic markers (*Mki67* and *Stmn1*) and exhaustion markers (*Pdcd1*, *Tigit*, and *Havcr2*), indicating chronic antigen-driven hyperproliferation and dysfunction. Conversely, combination therapy responders exhibited increased frequencies of T_naive_, *Gzmk*^+^ T_rm_, and T_emra_ cells, with the latter two subsets enriched for genes associated with robust cytotoxic and migratory function (*Cx3cr1*, *Itgb1*, and *Ccl5*) (Figures 7B and 7C). These shifts may stem from mBH3@NPs-mediated activation of intrinsic apoptosis pathways, thereby driving both compositional and phenotypic transitions within the CD8^+^ T cell compartment toward less differentiated, more functional subsets. Surprisingly, mBim@NPs further induced a significant expansion of innate-like T cells with cytotoxic potential (ILTCKs) when combined with T cells (Figures 7B and 7C), which is a tumor-resident innate-like CD8^+^ subtype exhibiting broad reactivity to unmutated self-antigens^58^^,^^61^ (Figures 7B and 7C). This expansion may be driven by an enhanced conventional dendritic cell (cDC)-mediated antigen presentation cascades, triggered by the robust ICD induced by mBim@NPs.^62^ Moreover, the combination of mBH3@NPs and ACT reprogramed exhausted and hyperproliferative CD8^+^ T cells toward a progenitor-like stemness phenotype characterized by reduced cytotoxicity (Figures 7E and 7F). Differential expression analysis revealed upregulation of cytotoxicity-related genes (*Gzmm* and *Gzma*), memory markers (*Sell*, *Tcf7*), and transcription factors responsive to effective TCR signaling (*Klf2*, *Fos*, and *Jun*), along with downregulation of immunosuppressive genes (*Pdcd1* and *Tigit*) within the TME compared to ACT alone (Figure 7G). These transcriptional shifts were primarily driven by tumor antigen exposure facilitated by APCs, particularly type I cDCs.^63^ Collectively, these results demonstrated that combination therapy reprogramed the transcriptional landscape of CD8^+^ T cells, driving their functional and phenotypic reconfiguration. Furthermore, the combination therapy indirectly remodeled the CD4^+^ T cell landscape via bystander effects (Figures S12A and S12B). Notably, treatment reduced dysregulated T_reg_ while increasing both the proportion and clonal diversity of functional CD4^+^ T_rm_ (Figures S12B and S12C), suggesting a more diverse, durable, and functionally competent CD4^+^ T cell population within the TME.

Integration of paired TCR data revealed distinct repertoire diversity pattern across treatments. Combination therapy with mPuma@NPs (TP) and mBim@NPs (TB) enhanced TCR diversity within key CD8^+^ subsets, particularly *Gzmk*+ T_rm_ and T_naive_ subsets, indicating broader recruitment of the endogenous T cell repertoire^64^ (Figure 7H). Overall, mBH3@NPs combinations (especially mBim@NPs) markedly broadened the CD8^+^ T cell TCR repertoire diversity rather than CD4^+^ T cells, as reflected by elevated Shannon entropy (Figure 7I). Normalized Shannon entropy analysis across subsets confirmed a more balanced TCR diversity distribution in combination groups, with significant increases in T_ex_ and proliferating CD8^+^ subsets (Figure 7J), which exhibited lower diversity and oligoclonality, reflecting tumor-antigen specificity under ACT monotherapy (Figure 7J). These observations suggest that combination mitigates clonal dominance of antigen-reactive subsets, broadening both the clonal breadth and functional heterogeneity of the antitumor T cell repertoire. Collectively, all these analyses revealed that the combination therapy drove CD8^+^ T cells toward memory-like states with expanded TCR diversity.

## Discussion

In this study, we demonstrated that mRNA LNP-mediated mtApoptosis induces effective antitumor immunity and synergistically enhances the therapeutic efficacy of ACT. We mainly evaluated two formulations derived from activator-type BH3-only proteins (mPuma@NPs and mBim@NPs), which preferentially induced ICD in cancer cells, thereby alleviating the immunosuppressive TME. Combined treatment with mBH3@NPs and ACT yielded synergistic antitumor effects across several solid tumor models. scRNA-seq analyses further revealed that this combinatorial strategy facilitated T cell trafficking, alleviated T cells dysfunction, and boosted cytotoxicity. Collectively, this study not only implements a combinatorial mRNA-based strategy but also provides mechanistic insights for augmenting T cell killing through mtApoptosis priming.

For clinical translation, BH3-domain-mediated mRNA therapy represents an effective strategy, whether as monotherapy or in combination with other immunotherapies, to overcome challenges currently faced by mtApoptosis-based therapeutics in solid tumors, including BH3 mimetics and BH3-derived peptides.^17^^,^^19^ These therapeutic mRNAs offer several key advantages. Specifically, they exhibit broad-spectrum binding capabilities to neutralize multiple pro-survival BCL-2 proteins,^32^^,^^33^ thereby intrinsically bypassing resistance driven by upregulation of non-targeted pro-survival proteins.^65^ Additionally, they function as direct activators to initiate robust mtApoptosis cascades. Furthermore, the mRNA modality itself possesses inherent immunostimulatory properties, functioning as a self-adjuvant to remodel the TME.^29^^,^^30^ Notably, combining mBH3@NPs with CTLs at lower E:T ratios achieved cytotoxicity compared to using CTLs alone with higher E:T ratios, indicating a shift in the E:T balance in favor of effector T cells. This may reduce the required T cell dose, lowering the cost and complexity of ACT in clinical applications. Importantly, careful timing of this combinatorial administration is crucial, as it helps not only to define an optimal therapeutic window but also to protect transferred T cells from potential mBH3@NPs-induced toxicity.^11^^,^^66^

Previous studies have shown that priming mtApoptosis can synergize with ACT to elicit more durable antitumor responses than either treatment alone.^8^^,^^9^^,^^10^^,^^11^^,^^67^ Nevertheless, the underlying mechanisms remain elusive. This synergy may rely on activation of the intrinsic apoptosis pathway, which amplifies sublethal damage accumulation arising from effector-target interactions.^9^^,^^11^^,^^68^ Notably, this effect is more pronounced at lower E:T ratios, where weaker or less frequent T cell cytotoxicity is sufficient to push cancer cells over the “apoptotic cliff”.^11^ Our *in vitro* data largely align with these findings, highlighting that mRNA LNP-mediated mtApoptosis priming provides a physiologically relevant approach to amplify death signals in solid tumors, where tumor-reactive CTLs often encounter a lower E:T ratio due to higher tumor burden. More importantly, our study revealed a layer of synergy achieved through immune landscape modulation. Specifically, pre-dosing of mBH3@NPs ameliorated the immunosuppressive TME, thereby creating a more permissive “battlefield” for subsequent ACT. It promoted trafficking of both endogenous and transferred effector T cells and enhanced their polyfunctionality, including improved cytotoxicity and alleviation of exhaustion. The predominant enhancement of effector function was observed in endogenous T cells rather than transferred T cells, which attributed to the intrinsic apoptotic effects directly induced by *in situ* mBH3@NPs administration. Meanwhile, highly proliferative metastatic T cells undergo a shift toward a stem-like/memory phenotype, potentially driven by a more immunologically permissive niche. Despite a reduced proportion of highly proliferative, clonally expanded T cells, this subtype exhibited a restoration from a highly exhausted state to a more sustained, surveillance-capable state. This phenotypic shift may initiate a positive feedback loop that supports long-term T cell persistence and durable antitumor immunity.^69^^,^^70^

Additionally, our findings revealed differential efficacy profiles of mBH3@NPs derived from distinct BH3-only family proteins (PUMA, BIM, NOXA, and BAD). Among these, domains from activator-type proteins (PUMA and BIM) induced stronger mtApoptosis as monotherapy, likely due to their broad binding affinity for multiple pro-survival BCL-2 proteins and their ability to directly activate BAX/BAK.^33^^,^^39^^,^^71^ In contrast, domains derived from sensitizer-type proteins (NOXA and BAD) exhibited weaker pro-apoptotic effects, potentially due to limited target engagement and subcellular mislocalization.^41^ Further optimization may focus on membrane-targeting engineering to enhance their functional activity. Notably, mBim@NPs induced more robust ICD and stronger immunostimulatory response compared to mPuma@NPs both *in vitro and in vivo*. This was associated with enhanced dendritic cell maturation, improved antigen presentation, and more effective alleviation of the immunosuppressive TME, thereby contributing to superior antitumor efficacy. Notably, scRNA-seq analyses revealed that mBim@NPs combined with ACT promoted ILTCK recruitment, potentially driven by increased IL-15 secretion from antigen-presenting cells (e.g., cDCs),^61^ thereby facilitating the differentiation of T cells into long-lived, high-avidity memory states.^72^^,^^73^ These results underscore the therapeutic potential of encoding direct activators like BIM or tBID, which broadly target anti-apoptotic proteins. However, for certain solid tumors dependent on specific pro-survival proteins (e.g., MCL-1),^74^ domains from other BH3-only proteins (NOXA) may offer greater selectivity and efficacy. Dynamic BH3 profiling may enable personalized therapeutic design by predicting tumor-specific sensitivity to BH3-domain-mediated apoptosis.^46^^,^^75^^,^^76^ We also observed that the therapeutic efficacy varied substantially between different tumor models. Compared to the MC-38 model, the superior response observed in the melanoma models may stem from their distinct dependencies on specific anti-apoptotic BCL-2 family proteins. The melanoma exhibits high reliance on BCL-2,^77^ potentially making them more vulnerable to BH3-domain-induced apoptosis. Colorectal cancers, including MC-38 model, exhibit stronger dependence on BCL-x_L_ and MCL-1,^78^^,^^79^ a redundant anti-apoptotic profile that remains ineffectively addressed by the current formulation. Additionally, despite being classified as an immunologically “cold” tumor, melanoma often exhibits a high tumor mutational burden (TMB).^80^, which enhances the release of diverse neoantigens upon mBH3@NPs-induced immunogenic cell death, thereby effectively priming robust anti-tumor immune responses.

### Limitations of the study

Despite these encouraging findings, further efforts are needed to address limitations that hinder clinical translation. The single-course combination of mBH3@NPs with adoptive T cells effectively delayed tumor progression and remodeled the immune microenvironment, it did not achieve complete tumor eradication. This outcome aligns with the well-recognized challenge of inducing sustained responses in solid tumor immunotherapy, which is often hindered by obstacles such as tumor heterogeneity and immune evasion. The present findings further suggest that repeated or sustained treatment regimens may be required to translate immune modulation into durable tumor clearance. Furthermore, this study primarily employed epitope-defined tumor models (B16-F10 and B16-OVA) and classical immunological tools (PMEL and OT-1). Further validation across diverse tumor types and alternative ACT modalities (CAR-T and TILs) is warranted. Although intratumoral administration is clinically feasible for certain surface-accessible tumors (e.g., melanoma) and some internal tumors (e.g., breast, colorectal cancers),^81^ future efforts should prioritize enhancing the targeting specificity of delivery systems.^82^

## Resource availability

### Lead contact

Further information and requests for resources and reagents should be directed to and will be fulfilled by the lead contact, Yongfeng Jin (jinyf@zju.edu.cn).

### Materials availability

This study did not generate new unique reagents.

### Data and code availability

The raw sequence data of bulk RNA-seq data generated in this study have been deposited in the Sequence Read Archive (SRA) database with accession numbers SRA: PRJNA1268331. The raw sequence data of single-cell RNA-seq and TCR-seq data have been deposited in the SRA database with accession number SRA: PRJNA1269318. All data needed to evaluate the conclusions are present in the paper and/or the supplemental information. This paper does not generate and report code. Any additional information required to reanalyze the data reported in this work paper is available from the lead contact upon request.

## Acknowledgments

This work was supported by research grants from the 10.13039/501100012166National Key Research and Development Program of China (2023YFC2604300), the Major Research plan of the 10.13039/501100001809National Natural Science Foundation of China (92581117), the “10.13039/100004502Pioneer” and “Leading Goose” R&D Program of Zhejiang (no. 2025C01138), the 10.13039/501100004731Natural Science Foundation of Zhejiang Province (LZ25C050001), the 10.13039/501100012226Fundamental Research Funds for the Central Universities (no.K20220228), and the Starry Night Science Fund at Shanghai 10.13039/100005235Institute for Advanced Study of 10.13039/501100004835Zhejiang University (SN-ZJU-SIAS-009). We thank members of the Jin laboratory for suggestions and discussion during the course of this work.

## Author contributions

Y.J. contributed to the conception of this project. J.F., Y.L., Z.Z., B.C., L.R., Z.G., Y.M., and Y.J. contributed to the design and the experiments. J.F., Y.L., Z.Z., and N.B. contributed to the data analyses. Y.J., J.F., H.D., R.P., J.W., and Y.M. contributed to the writing of the manuscript. All the authors contributed the results and gave comments and discussion on the manuscript.

## Declaration of interests

The authors declare no conflict of interest.

## Declaration of generative AI and AI-assisted technologies in the writing process

During the preparation of this work, the author(s) did not use generative AI and AI-assisted technologies.

## STAR★Methods

### Key resources table

**Table:** 

REAGENT or RESOURCE	SOURCE	IDENTIFIER
**Antibodies**		

Bax antibody	Cell Signaling Technology	Cat#2772; RRID:AB_10695870
Bak antibody	Cell Signaling Technology	Cat#12105; RRID:AB_2716685
Bcl-2 (D17C4) Rabbit mAb	Cell Signaling Technology	Cat#3498; RRID:AB_1903907
Puma (E2P7G) Rabbit mAb	Cell Signaling Technology	Cat#98672; RRID:AB_3096180
Bim (C34C5) Rabbit mAb	Cell Signaling Technology	Cat#2933; RRID:AB_1030947
Caspase-9 Antibody	Cell Signaling Technology	Cat#9504; RRID:AB_2275591
Caspase-3 (D3R6Y) Rabbit mAb	Cell Signaling Technology	Cat#14220; RRID:AB_2798429
Cleaved-Caspase 3 (Asp175), p17 Antibody	Abmart	Cat#TA7022S; RRID:AB_2936835
Cleaved-Caspase 9 (Asp353) Antibody	Abmart	Cat#TA5240S; RRID:AB_3740854
Bcl-XL Recombinant Rabbit Monoclonal Antibody	Huabio	Cat#ET1603-28; RRID:AB_2924969
MCL1 Recombinant Rabbit Monoclonal Antibody	Huabio	Cat#ET1606-14; RRID:AB_3069723
Anti-beta Actin antibody	Abcam	Cat#ab8224; RRID:AB_449644
Calreticulin Polyclonal antibody	Proteintech	Cat#27298-1-AP; RRID:AB_2880835
HRP, Goat Anti-Rabbit IgG(H + L)	Earth	Cat#E030120; RRID:AB_3073916
Glut1 (E4S6I) Rabbit mAb	Cell Signaling Technology	Cat#73015; RRID:AB_3064908
Dylight 488-goat anti-rabbit igG	Earth	Cat#060812; RRID:AB_3065583
Anti-Cytochrome c	Biolegend	Cat#612304; RRID:AB_2090159
PE Anti-mouse CD3	Biolegend	Cat#100206; RRID:AB_312663
Alexa Fluor® 700 Anti-mouse CD3	Biolegend	Cat#100216; RRID:AB_493697
FITC Anti-mouse CD4	Biolegend	Cat#100406; RRID:AB_312691
PerCP Anti-mouse CD8a	Biolegend	Cat#100732; RRID:AB_893423
Brilliant Violet 510™ Anti- mouse CD90.1 (Thy1.1)	Biolegend	Cat#202535; RRID:AB_2562643
APC Anti-mouse CD90.2 (Thy1.2)	Biolegend	Cat#140312; RRID:AB_10640728
FITC Anti-mouse CD80	Biolegend	Cat#104706; RRID:AB_313127
APC Anti-mouse CD86	Biolegend	Cat#105011; RRID:AB_493343
PE Anti-mouse CD11c	Biolegend	Cat#117308; RRID:AB_313777
PerCP/Cyanine5.5 Anti-mouse CD206	Biolegend	Cat#141716; RRID:AB_2561992
PE Anti-mouse CD11b	Biolegend	Cat#101208; RRID:AB_312791
APC Anti-mouse Ly-6G/Ly-6C (Gr-1)	Biolegend	Cat#108412; RRID:AB_313377
APC Anti-mouse F4/80	Biolegend	Cat#123116; RRID:AB_893481
Pacific Blue™ Anti-mouse CD80	Biolegend	Cat#104724; RRID:AB_2075999
Alexa Fluor 700 Anti-mouse IFN-γ	Biolegend	Cat#505824; RRID:AB_2561300
APC Anti-mouse CD45	Biolegend	Cat#147708; RRID:AB_2563540
FITC Anti-mouse NK1.1	Biolegend	Cat#156508; RRID:AB_2876526
Brilliant Violet 510™Anti-mouse CD25	Biolegend	Cat#102041; RRID:AB_2562269
Alexa Fluor 700 Anti-mouse FOXP3	Biolegend	Cat#126422; RRID:AB_2750493
PE Anti-mouse Granzyme B	Biolegend	Cat#372208; RRID:AB_2687032
FITC Anti-mouse Perforin	Biolegend	Cat#154309; RRID:AB_2910315
Pacific Blue™ Anti-mouse CD45	Biolegend	Cat#157212; RRID:AB_2876534
Brilliant Violet 510™ Anti-mouse CD3	Biolegend	Cat#100233; RRID:AB_2561387
FITC Anti-mouse CD279	Biolegend	Cat#135214; RRID:AB_10680238
APC Anti-mouse CD223	Biolegend	Cat#125210; RRID:AB_10639727
Brilliant Violet 510™ Anti-mouse CD62L	Biolegend	Cat#104441; RRID:AB_2561537
FITC Anti-mouse CD44	Biolegend	Cat#156007; RRID:AB_2941437
APC/Cyanine7 Anti-mouse CD69	Biolegend	Cat#104525; RRID:AB_10683447
Alexa Fluro® 488 Anti-HA.11 Epitope Tag Antibody	Biolegend	Cat#901509; RRID:AB_2565072

**Chemicals, peptides, and recombinant proteins**		

SM-102	AVT	Cat#2089251-47-6
DMG-PEG2000	AVT	Cat#160743-62-4
DSPC	AVT	Cat#816-94-4
CHO-HP	AVT	Cat#57-88-5
Solar Fluor 647	Solarbio	Cat#S1067
ABT-199	Medchem Express	Cat#HY-15531
Phosal 50 PG	Fisher Scientific	Cat#NC0130871
PEG400	Sigma-Aldrich	Cat#202398
Recombinant Murine IL-2	Peprotech	Cat#212-12
Recombinant human IL-2	Peprotech	Cat#200-02
Recombinant Murine GM-CSF	Peprotech	Cat#315-03
Recombinant Murine IL-4	Peprotech	Cat#214-14
RetroNectin	Takara	Cat#T100A
hgp100(25–33)	GenScript	Cat#RP20344
OVA (257–264), amide	Solarbio	Cat#CLP0705

**Critical commercial assays**		

EasyCap T7 Co-transcription Kit with CAG trimer	Vazyme	Cat#DD4203-01
EasyPure RNA Purification Kit	Transgen	Cat#ER701
Annexin V-FITC Apoptosis Detection Kit	Solarbio	Cat#CA1020
CFDA,SE Cell Proliferation And Tracer Assay Kit	Solarbio	Cat#CA1200
2-NBDG Glucose Uptake Assay Kit	Solarbio	Cat#G9860
Reduced Glutathione(GSH)Content Assay Kit	Solarbio	Cat#BC1175
Oxidized Glutathione(GSSG)Content Assay Kit	Solarbio	Cat#BC1180
Reactive Oxygen Species Assay Kit	Meilunbio	Cat#MA0219
Mitochondrial Membrane Potential Assay Kit with JC-1	Solarbio	Cat#M8650
Enhanced ATP Assay Kit	Beyotime	Cat#S0027
ElaBoX™ Mouse HMGB1 ELISA Kit	Solarbio	Cat#SEKM-0145
Mouse CD8+T cell Isolation Kit	Selleck	Cat#B90011
Human CD8^+^ T cell Negative Selection	MedChemExpress	Cat#HY-KO351
Dynabeads Mouse T-Activator CD3/CD28	Thermo fisher	Cat#11456D
Dynabeads Human T-Activator CD3/CD28	Thermo fisher	Cat#11161D

**Deposited data**		

Bulk RNA-seq	Generated by the Authors	PRJNA1268331
Single-cell RNA-seq and TCR-seq	Generated by the Authors	PRJNA1269318

**Experimental models: Cell lines**		

B16-F10	Hai Xing Biosciences	RRID:CVCL_0159
B16-OVA	a gift from Y. Ping	N/A
CT-26	Hai Xing Biosciences	RRID:CVCL_7256
MC-38	a gift from H. Zhu	RRID:CVCL_B288
bEnd3	Hai Xing Biosciences	RRID:CVCL_0170
HEK-293T	Hai Xing Biosciences	RRID:CVCL_0063
HeLa	Hai Xing Biosciences	RRID:CVCL_0030

**Experimental models: Organisms/strains**		

Mice: C57BL/6J	The Jackson laboratory	RRID:IMSR_JAX:000664
Mice: C57BL/6-Tg(TcraTcrb)1100Mjb/J	The Jackson laboratory	RRID:IMSR_JAX:003831
Mice: B6.Cg-Thy1^a^/Cy Tg(TcraTcrb)8Rest/J	The Jackson laboratory	IMSR_JAX:005023
Mice: NOD.Cg-Prkdc^scid^ Il2rg^tm1Wjl^/SzJ	The Jackson laboratory	RRID:IMSR_JAX:005557

**Recombinant DNA**		

psPAX2 Plasmid	A gift from X. Zhang	N/A
pMD2.G Plasmid	A gift from X. Zhang	N/A
NY ESO 1 Ag Luci ZsGreen	A gift from R. Pan	N/A
NY-ESO-1 TCR	A gift from R. Pan	N/A

**Software and algorithms**		

ImageJ/Fiji		https://imagej.nih.gov/ij
Prism (version 8.0.2)	GraphPad	https://www.graphpad.com/features
Flowjo Software v10.8.1.	Flowjo, LLC	https://www.flowjo.com/solutions/flowjo
ZEN	ZISS	https://www.zeiss.com/microscopy/en/products/software/zeiss-zen.html

### Experimental model and study participant details

#### Materials and reagents

RPMI 1640 basic, Dulbecco’s modified Eagle’s medium (DMEM), fetal bovine serum (FBS) and penicillin streptomycin were purchased from Thermo Fisher Scientific (USA). SM-102, cholesterol CHO-HP, DSPC, and DMG-PEG2000 was purchased from AVT Co. Ltd., Shanghai, China. NY-ESO-1-Ag-Luci-ZsGreen and NY-ESO-1 TCR plasmids were gifts from R. Pan (Zhejiang university). psPAX2 Plasmid and pMD2.G Plasmid were gifts from X. Zhang (Zhejiang university).

#### Cell lines and animals

B16-F10, CT-26, bEnd3, HEK 293T, HeLa cells were purchased from Hai Xing Biosciences (Suzhou, China). B16-OVA cell was a gift from Y. Ping (Zhejiang university). MC-38 cell was a gift from H. Zhu (Zhejiang university). B16-F10, B16-OVA, MC-38, bEnd3, HEK 293T, HeLa were cultured in DMEM medium containing 10% fetal bovine serum (FBS) and 1% penicillin/streptomycin. CT-26 was cultured in RPMI 1640 medium containing 10% fetal bovine serum and 1% penicillin/streptomycin. Cells were tested to ensure no mycoplasma contamination.

Six-to eight-week-old C57BL/6 female mice (4–6 weeks old) were obtained from Qi Zhen laboratory animal Tech Co. Ltd., China and used for antitumor effect evaluation. Female 6-week-old NSG mice were obtained from Shanghai Model Organism Center, Inc and used for human tumor murine xenograft models.

Thy1.1^+^ pmel-1 (PMEL) mice with a transgenic TCR recognizing gp100_(25-33)_ and OT-1 mice with a transgenic TCR recognizing ovalbumin (OVA_257-264_) peptide were originally purchased from the Qi Zhen laboratory animal Tech Co. Ltd., China and maintained at the EPFL’s pathogen-free facility using for adoptive T cell therapy. The animals were randomly assigned into cages in a room maintained at 22 ± 2.0°C and 50 ± 10% humidity and subjected to a 12-h light/12-h dark cycle before the study. All mice were given access to a standard diet and tap water. All animal experiments were performed in accordance with the China Public Health Service Guide for the Care and Use of Laboratory Animals. The sex of all mice was confirmed by visual examination of the external genitalia. All animal experiment protocols were approved by the Animal Ethical Committee of Zhejiang University and use committee of Zhejiang university (ZJU20240885).

### Method details

#### Synthesis of mRNA

For BH3 mRNA, it encodes for domains from pro-apoptotic BH3-only proteins, including Bim, Puma, Bad and Noxa (Table S1). The BH3 domains were fused to Stefin A quadruple aptamer scaffold to anchor and present the BH3 domain structure and enable physiological function or not.^40^ The whole target fragments were then connected to linearized template vectors (Takara Biotech Co. Ltd., China) by seamlessly cloning to construct plasmids for downstream *in vitro* transcription reactions. mRNA was synthesized using standard *in vitro* transcription methods. Plasmids that encoded specific antigens were digested overnight using *HindIII* to obtain a linearized template, followed by the production of mRNA using EasyCap T7 Co-transcription Kit with CAG trimer (Vazyme Biotech Co. Ltd., China) as per manufacturer instructions. And then mRNA purified by EasyPure RNA Purification Kit (Transgen Biotech Co. Ltd., China). All mRNAs were analyzed by agarose gel electrophoresis and were stored frozen at −80°C.

#### Preparation and characterization of mBH3@NPs

The mBH3@NPs formulation was based on the disclosed prescription by Moderna.^83^ Specifically, ionizable lipid SM-102, cholesterol CHO-HP, DSPC, and DMG-PEG2000 (AVT Co. Ltd., Shanghai, China) mixed at a mass ratio of 50:10:38.5:1.5 with absolute ethanol. Three volumes of antigen-encoding mRNAs [1:25 (w/w) mRNA to lipid, nitrogen/phosphate (N/P) ratio = 6] were suspended in sodium citrate buffer (pH 4.5). Then the two solutions were mixed by rapid pipetting and transferred into dialysis overnight at 4°C against PBS. Particles in PBS were used for further characterization. The size distribution and zeta potential of mBH3@NPs was determined by dynamic light scattering (DLS) (Zetasizer Nano ZS90, Malvern Instruments, Malvern, UK). The morphology was assessed using Transmission Electron Microscope (TEM). To verify the stability of the mRNA NPs formulation, the diameters were assessed after incubated at both room temperature and 4°C for various hours.

#### *In vitro* cytotoxicity evaluation

B16-F10, CT-26, HEK 293T and bEnd3 cells were inoculated in 96-well plates at a density of 1×10^4^, respectively, and cultured with medium containing 10% fetal bovine serum for 24 h. Then incubate for 24 h using mBH3@NPs at concentrations of 0, 1, 10, 25, 50, 100, 250, 500 and 1,000 ng mL^−1^, respectively. Finally, added 10 μL solution from Cell-Counting-Kit-8 (APExBIO Tech, China) to each well and cell viability was detected at 450 nm after incubation for 1 h.

#### Apoptosis assay

Cancer cells were treated with different dosage of mBH3@NPs for 12 h. After treatment, cells were collected, stained with FITC-conjugated Annexin V and PI at 4°C for 10 min, and analyzed by flow cytometry (CytoFLEX, Beckmancoulter, US). Data were analyzed by Flowjo software v10.8.1.

#### Western blot analysis

B16-F10 cells were seeded in a 6-well plate at a density of 5 × 10^5^ cells per well. After cells reached 70–80% confluence, cells were treated with NPs, naked mRNA, mPuma@NPs and mBH3@NPs. Protein samples were extracted from cells lysed with radioimmunoprecipitation (RIPA) buffer supplemented with protease inhibitors. Protein samples were separated by sodium dodecyl sulfate polyacrylamide gel electrophoresis (SDS-PAGE), transferred to polyvinylidene fluoride (PVDF) membranes and then blocked with 5% BSA. Diluted primary antibody of apoptosis pathway including Bax, Bcl-2, Bcl-x_L_, Mcl-1, Caspase-3, Cleaved-Caspase3, Caspase-9, Cleaved-Caspase9 was incubated with the membranes overnight, followed by incubation with secondary antibody for 2 h at 37°C. After washing three times with TBST (20 mM Tris, 160 mM NaCl, 0.1% Tween 20), the ChemiDoc XRS system (Bio-Rad) was used to detect the chemiluminescent signals. Beta-actin was used as a housekeeping control.

#### Glucose uptake assay

Glucose uptake was assessed by flow cytometry using the fluorescent glucose analog 2-NBDG (Solarbio Science Technology Co., Ltd.) and Glut-1 Rabbit mAb (CST, 73015). Briefly, pre-activated OT-1 CD8^+^ T cells were co-cultured with tumor cells that had been pre-treated with either mPuma@NPs, mBim@NPs, or untreated. After co-culture, T cells were harvested and incubated for dual analysis: Cells were incubated with 2-NBDG for 1 h at 37°C; Cells were stained with Glut-1 mAb followed by Dylight488-conjugated secondary antibody. The percentage of 2-NBDG-positive and Glut-1-positive T cell populations was determined by flow cytometry for each group.

#### Glutathione measurement

Glutathione and oxidized glutathione (GSSG) levels were determined using GSH/GSSG Assay Kit from Solarbio. Briefly, pre-activated OT-1 CD8^+^ T cells were cocultured with tumor cells that had been pre-treated with either mPuma@NPs, mBim@NPs, or untreated. For each group, 2 × 10^6^ T cells were lysed in 200 μL of the provided lysis buffer. After centrifugation at 12, 000 × g for 10 min at 4°C, the supernatant was collected for analysis. A standard curve was prepared using standards. The absorbance at 412 nm was measured, and the concentrations of total glutathione and GSSG were determined accordingly, the GSH/GSSG ratio were subsequently derived.

#### ROS, JC-1, ATP and HMGB1 detection

CT-26 and B16-F10 cells (2×10^5^ per well) were seeded in a 12-well plate, treated with PBS, NPs, mPuma@NPs, and mBim@NPs (1 μg mL^−1^) for 12 h. The level of ROS and mitochondrial membrane potential in cells were detected using the ROS Assay Kit and Mitochondrial membrane potential assay kit with JC-1 (Solarbio Science Technology Co., Ltd.), respectively. The cell culture medium was collected for extracellular ATP assay (Beyotime Biotechnology) and HMGB1 analysis (Solarbio Science Technology Co., Ltd.). The cell lysate was collected for intracellular ATP assay and HMGB1 analysis.

#### Cell immunofluorescent staining

B16-F10 and CT-26 cells were seeded on glass slides in 6-well plates and were treated with PBS, NPs, mPuma@NPs, and mBim@NPs (1 μg mL^−1^) for 12 h. After that, cells were washed twice with PBS fixed with 4% paraformaldehyde for 20 min at room temperature. Then cells were permeabilized with 0.1% Triton X-100 PBS (PBST) and blocked with a blocking solution for 1 h at room temperature. Next, the cells were incubated in the diluted CRT antibody (1:200) overnight at 4°C. Then cells were washed with PBS three times and incubated in secondary antibody with dylight488 for 2 h at temperature. The, cells were washed with PBS three times and stained with 4′,6-diamidino-2-phenylindole (DAPI) for 10 min and imaged using CLSM (Leica FLUOVIEW FV3000 CLSM, Olympus, Japan).

#### *In vitro* stimulation of dendritic cells

BMDCs were isolated from the bone marrow of female C57BL/6 mice as described previously.^84^ Briefly, single-cell suspensions were prepared, passed through 45 μm cell strainers, and cultured in RPMI 1640 complete medium supplemented with 20 ng mL^−1^ GM-CSF and 10 ng mL^−1^ IL-4 (Peprotech) for one week. B16-F10 cells were pretreated with PBS (Control), NPs, mPuma@NPs, and mBim@NPs for 12 h. Following this, co-culturing with BMDCs for 48 h. The maturation biomarkers for DCs (CD80 and CD86) were assessed by flow cytometry, gated on CD11c^+^ cells.

#### Preparation and activation of PMEL and OT-1 CD8^+^ T cells

The spleen of PMEL and OT-1 mice was sterilized in the biosafety cabinet and then mechanically meshed through a 45 μm strainer to obtain a single-splenocyte suspension. After lysis with red blood cell lysate buffer for 3 min, splenocytes were resuspended with DPBS. Using CD8a^+^ T cell Isolation Kit (Selleck) to obtain purified CD8^+^ T cell suspension. After that, resuspended 1×10^6^ purified naive T cells in a 24-well plate with RPMI medium supplemented with 5 mM HEPES, 2 mM Glutamax, 50 μg/mL Pen/Strep, 5 mM NEAA, 5 mM sodium pyruvate, 10% certified heat-inactivated FBS, 50 μM beta-mercaptoethanol, as well as 30 IU mL^−1^ murine IL-2 (Peprotech), hpg100_25-33_ (Genscript) or OVA_257-264_ (Solarbio) for activation. Add 25 μL Dynabeads Mouse T-activator CD3/CD28 magnetic beads (ThermoFisher Scientific Inc.) and incubate T cells at 37°C for 72 h. After stimulation, cells were pooled into flasks for expansion. Half of the medium was replaced with fresh medium to keep the cell density about 2×10^6^ cells mL^−1^ for another 72-h incubation.

#### Isolation of human CD8^+^ T cells

Human CD8^+^ T cells were isolated from PBMCs using the Human CD8^+^ T cell Negative Selection Kit (MedChemExpress, #HY-KO351). Enriched cells were cultured in RPMI-1640 medium supplemented with 5 mM HEPES, 2 mM Glutamax, 50 μg/mL Pen/Strep, 5 mM NEAA, 5 mM sodium pyruvate, 10% certified heat-inactivated FBS, 50 μM beta-mercaptoethanol, as well as 30 IU/mL of human IL-2 (Proteintech). Cells were activated in a 48-well plate at 2×10^6^/mL with CD3/CD28 Dynabeads (ThermoFisher Scientific, #11161D) (bead: cell = 1:1), then transferred to flasks and maintained at 1×10^6^ cells/mL with semi-medium changes every 2–3 days.

#### Lentiviral transduction

NY-ESO-1-specific TCR lentivirus was packaged in HEK-293T cells using the third-generation packaging plasmids psPAX2 and pMD2.G. Viral supernatant was harvested 48 and 72 h post-transfection, concentrated, and tittered. Non-tissue-culture-treated 24-well plates were coated overnight at 4°C with RetroNectin (25 μg/mL; Takara), blocked with 2% BSA, and washed. Lentiviral particles were added and centrifuged onto the plate (2,000 g, 2 h, 32°C). After removing the supernatant and washing, 0.5 × 10^6^ activated CD8^+^ T cells were added per well in medium containing 10 μg/mL protamine sulfate. Plates were centrifuged (500 g, 20 min) and incubated for 72 h. Cells were harvested, stained with anti-HA antibody (#901509, BioLegend), and FACS-sorted to purify TCR-positive populations. Sorted T cells were maintained in complete medium with 30 IU/mL IL-2, split as needed to keep density between 1× 10^6^ cells/mL.

#### Generation of NY-ESO-1-expressing HeLa cells

NY-ESO-1 antigen-expressing HeLa cells were generated by lentiviral transduction. Lentivirus was produced by co-transfecting HEK-293T cells with the transfer plasmid (NY-ESO-1-Ag-Luci-ZsGreen), psPAX2 and pMD2.G packaging plasmids using NB transfection reagent (#NB02). Viral supernatant was collected 48 and 72 h post-transfection. For infection, HeLa were resuspended in the virus stock (5 × 10^5^ cells/mL) and centrifuged (1,000 g, 45 min, 30°C). After a second round of virus addition and centrifugation, cells were incubated for 1 h at 37°C, washed twice to remove polybrene, and returned to culture. After approximately two doubling periods, ZsGreen-positive cells were isolated by fluorescence-activated cell sorting (FACS).

#### *In vitro* CD8^+^ T cell killing

B16-OVA cells were seeded into a 24-well plate at a density of 7×10^4^ cells per well overnight. Then cells were treated with 500 μL of medium containing different doses of mBH3@NPs for 12 h incubation, and different E:T ratios of activated OT-1 CD8 T cells for another 24-h incubation. After incubation, tumor cells were collected and analyzed using ANNEXIN V-FITC/PI kit (Solarbio Science Technology Co., Ltd.). CompuSyn software was used to calculate the combination index (CI at ED_50_) if there is a synergy of mBH3@NPs and T cells. The percentage of killing efficiency was calculated as 100%-Normalized cells (Annexin V^−^ PI^−^) %. Cell supernatants were collected to evaluate IFN-γ and Granzyme B using ELISA kit (Solarbio Science Technology Co., Ltd.). The CD8^+^ T cells were collected for flow cytometry to evaluate IFN-γ, GrB, PD-1, and LAG-3.

#### Tumor models

For mBH3@NPs administration, female C57BL/6 mice aged 6–8 weeks were subcutaneously injected with B16-F10 or MC-38 cell line 1×10^6^ in 200 μL PBS. Once the tumors reached approximately 50–100 mm^3^, intratumorally injected with saline, empty NPs, mPuma@NPs, and mBim@NPs (10 μg per mouse) as shown in the timeline in Figure 6A. For mice treated with ABT-199, the compound was prepared in a vehicle consisting of 10% ethanol, 30% polyethyleneglycol-400, and 60% phosal 50 propylene glycol. ABT-199 were administrated at the doses and schedules indicated in Figure S10. For the combination therapy of mBH3@NPs and ACT therapy, female C57BL/6 mice aged 6–8 weeks were injected subcutaneously with 2 × 10^6^ B16-OVA or B16-F10 melanoma cells. Once the tumors reached approximately 50–100 mm^3^, intratumorally injected with saline, NPs, mPuma@NPs, and mBim@NPs. One day later, the pre-activated CD8^+^ T cells (2×10^6^ in 100 μL PBS) from OT-1 mice or PMEL mice were intravenously injected into the caudal vein of the tumor-bearing mice, respectively. Tumor lengths and widths were determined every 2–3 days by digital caliper measurement and tumor volume was calculated using the following formula: tumor volume = (length × width^2^)/2. For all studies, the mice were euthanized once the tumors reached a diameter of 15 mm.

For tumor murine xenograft models, female 6-week-old NSG mice (Shanghai Model Organism Center, Inc) were subcutaneously injected with 4×10^6^ HeLa-NY-ESO-1 cells resuspended in a 50:50 mixtures of cold PBS and Matrix (Vazyme Biotech Co. Ltd., China). Once the tumors reached approximately 50–100 mm^3^, the mice were randomly assigned to four groups and intratumorally injected with mPuma@NPs and mBim@NPs. One day later, NY-ESO-1 TCR transduced CD8^+^ T cells (2×10^6^ in 100 μL PBS) were intravenously injected. Tumor lengths and widths were determined every 2–3 days by digital caliper measurement and tumor volume was calculated using the following formula: tumor volume = (length × width^2^)/2.

#### Migration assay of T cells

For *in vitro* Transwell migration assay of T cells, mice bearing approximately 100 mm^3^ B16-OVA tumor cells were treated with saline, mPuma@NPs, and mBim@NPs (10 μg per mouse). On the second day, tumors were collected and directly ground over 45-μm strainer and supplemented with complete DMEM culture (5×10^5^ mL^−1^). The cells were then centrifuged at 12,000×g for 30 min to obtain the tumor interstitial fluid. Seed 2 × 10^5^ B16-OVA cells with 400 μL diluted tumor interstitial fluid were placed at the bottom well, and 5 × 10^5^ isolated OT-1 T cells in 200 μL were stained with CFDA, SE (Solarbio Science Technology Co., Ltd.) for 30 min and then placed on the upper well (5 μm, Corning) for 24 h co-culture. Collect cells from bottom wells and added counting beads (BioLegend) for analyzing the migration percentage of OT-1 T cells. For *in vivo* migration assay of T cells, C57BL/6 mice were implanted with B16-OVA first, and once the tumors reached approximately 50–100 mm^3^, intratumorally injected with Saline, mPuma@NPs, and mBim@NPs. One day later, the pre-activated CD8^+^ T cells (2×10^6^) from OT-1 mice were stained with cell tracker (Solar Fluor 647, Solarbio Science Technology Co., Ltd.) and intravenously injected into the caudal vein of the tumor-bearing mice. After 6h, the fluorescence from the mice was imaged using the *In Vivo* Imaging System (IVIS Spectrum, Caliper, US).

#### Flow cytometry analysis

The tumors, spleens and lymph nodes were cut into pieces. For tumor tissues digestion, RPMI 1640 media containing 10% FBS, collagenase IV (1 mg mL^−1^), hyaluronidase (0.2 mg mL^−1^) and DNase (0.1 mg mL^−1^) were added for 20–30 min in 37°C water baths. Then tissue suspension was passed through a 45-μm filter membrane to form single-cell suspensions. After erythrolysis with 1×RBC buffer, cells were blocked with TruStain FcX (anti-mouse CD16/32) antibody for 20 min at 4°C, and then stained on the ice with fluorescence-conjugated antibodies for 30 min. After staining, cells were resuspended with 200 μL FACS buffer and ultimately evaluated by flow cytometry (CytoFLEX, Beckmancoulter, US) and analyzed using FlowJo (v10.8.1). Antibodies used in this study are listed in STAR Methods. Gating strategy for main immune cells in this study is shown in Figure S13.

#### Immunofluorescence and H&E staining

After tumor treatment, the mice were euthanized. Collect the tumor tissues and various organs (lung, heart, liver, kidney, and spleen) and fixed with 4% paraformaldehyde overnight. All organs were embedded in paraffin and sliced to a thickness of 5 μm. Then stained with H&E for detecting potential pathological changes. For immunofluorescence staining, samples were incubated with different fluorescently labeled antibodies (TUNEL, Ki67, CD8, CRT, IFN-γ, PD-1, CD44, CCR7, CD69, DAPI) and slides were imaged using a digital slicing scanner (3D Histech).

#### Multi-cytokine assay

Briefly, tumor tissue was homogenized, and the cell supernatant of each treatment group was collected for cytokine assay using ELISA kit from Solarbio Life Sciences (China). The selected cytokines included Granzyme B, TNF-α, IFN-γ, IL-6, IL-12. For multi-cytokine, tumor homogenate supernatants were assessed using ABplex Mouse Cytokine 15-Plex Assay Kit (ABclonal Biotechnology Co., Ltd.).

#### Blood and serological tests

To assure the biosafety of this therapy, we retrieved serum by centrifuging at 3,000 rpm for 20 min. Serum aspartate transaminase, alanine transaminase, total bilirubin, creatinine, urea concentrations were determined. Serum cytokines related to cytokine release syndrome (IL-6, IL-10, TNF-α, IFN-γ) were determined by ELISA kit from Solarbio Life Sciences (China).

#### Library construction and sequencing

Total RNA was extracted using TRIzol (Thermo Fisher, 15596018) following the manufacturer’s procedure. After total RNA was extracted, mRNA was purified from total RNA using Dynabeads Oligo (dT) (Thermo Fisher, CA, USA) with two rounds of purification. Following purification, the mRNA was fragmented into short fragments using divalent cations under elevated temperature (NEB, cat. e6150, USA). Cleaved RNA fragments were then reverse-transcribed to create cDNA using SuperScript II Reverse Transcriptase (Invitrogen, cat. 1896649, USA), which was subsequently used to synthesize U-labeled second-stranded DNAs with *E. coli* DNA polymerase I (NEB, cat.m0209, USA), RNase H (NEB, cat.m0297, USA), and dUTP Solution (Thermo Fisher, cat. R0133, USA). An A-base was added for ligation to the indexed adapters. Each adapter contained a T-base overhang for ligating the adapter to the A-tailed fragmented DNA. Dualindex adapters were ligated to the fragments, and size selection was performed with AMPureXP beads. After treatment with the heat-labile UDG enzyme (NEB, cat.m0280, USA), the ligated products were amplified. The average insert size for the final cDNA libraries was 300 ± 50 bp. Finally, we performed 2 × 150 bp paired-end sequencing (PE150) on an Illumina Novaseq 6000 (LC-Bio Technology CO., Ltd., Hangzhou, China). The cDNA libraries constructed were then sequenced run with Illumina Novaseq 6000 sequence platform. Using the Illumina paired-end RNA-seq approach, the transcriptome was sequenced, generating a total of millions of 2 × 150 bp paired-end reads. Reads were further filtered using Cutadapt (https://cutadapt.readthedocs.io/en/stable/). The sequence quality was verified using FastQC (http://www.bioinformatics.babraham.ac.uk/projects/fastqc/, 0.11.9) including the Q20, Q30, and GC-content of the clean data.

#### Single-cell dissociation

B16-F10 bearing mice were first treated with saline, mPuma@NPs, and mBim@NPs (10 μg per mice). One day later, the pre-activated CD8^+^ T cells (2×10^6^ in 100 μL PBS) from PMEL mice were intravenously injected into the caudal vein of the tumor-bearing mice. Tumor tissues were harvested six days later. Then a scRNA-seq was performed at the laboratory of Lc-Bio Technologies (Hangzhou) Co., Ltd. The obtained tumor tissues were minced into 0.5 mm^2^ pieces and digested in a dissociation solvent (0.35% collagenase IV, 2 mg mL^−1^ papain, 120 U mL^−1^ DNase I) for 20 min. Following digestion, the specimens were filtered through 70-micron cell sieves to achieve single cell suspensions. The cell suspensions were then treated with erythrocyte lysis buffer (MACS 130-094-183,10×) for 10 min. After lysis, the cells were centrifuged, and the cell precipitate were resuspended in flow cytometry buffer. Add 100 μL of Dead Cell Removal MicroBeads (MACS130-090-101) for 15 min incubation. At the end of incubation, binding buffer was added to MS Columns (130-042-201) to remove reagents. APC Anti-mouse CD45 (BioLegend, catalog no. 147708), PE Anti-mouse CD3 (BioLegend, catalog no. 100206) were employed to label the cell surface. The cells were labeled for 30 min at 4°C, protected from light. Subsequently, CD45^+^/CD3^+^ cells were extracted and analyzed from each specimen. Cell viability was assessed using trypan blue staining method, with a required viability of >85%. The number of cells was counted using a Countess II Automated Cell Counter, and the cell concentration was adjusted to 700–1200 cells μL^−1^.

#### Single-cell sequencing

Single-cell suspension was added to the 10× Chromium chip according to the instructions for the 10× Genomics Chromium Single-Cell 3′ kit (V3), with the expectation of capturing 8,000 cells. Libraries were run on the HiSeq4000 for Illumina sequencing. cDNA amplification and library construction were performed according to standard protocols. Libraries were sequenced by LC-Bio Technology (Hangzhou, China) on an Illumina NovaSeq 6000 sequencing system (double-end sequencing, 150 bp) at a minimum depth of 20,000 reads per cell.

#### Statistical analysis of single-cell RNA data

Results from Illumina sequencing offline were converted to FASTQ format using bcl2fastq software (version 5.0.1). The scRNA-seq data were compared to reference genome using CellRanger software, and cellular and individual cellular 3′ end transcripts were identified and counted in the sequenced samples (Cell-ranger, version 9.0.1). The output CellRanger expression profile matrix was loaded into Seurat (v3) for filtering low-quality cells from scRNA-seq data, followed by downscaling and clustering. Low-quality cells were filtered by retaining only cells with at least 500 total counts and more than 200 detected genes. Gene annotated as ribosomal (RPS, RPL), mitochondrial (MT-), or DnaJ/Hsp40 family members (DNAJ) were excluded from downstream analysis based on their gene symbols. For visualization purposes, we projected the obtained latent embedding onto a two-dimensional space using UMAP function. DEGs within each cell cluster was calculated by the scanpy.tl.rank_genes_groups function (method = ‘*t* test’). To assess the diversity of the TCR repertoire, we utilized Shannon entropy calculation using the scirpy.tl.alpha_diversity function.

### Quantification and statistical analysis

All experiments were repeated at least three times. Statistical data were graphed and analyzed using GraphPad Prism version 8.0.2 (GraphPad Software, Inc., USA) and ImageJ. Quantitative results are presented as means ± SDs. Sample sizes for each statistical analyses were noted in the figure legends. For comparisons between two groups, a two-tailed unpaired Student’s *t* test was employed. Comparisons among multiple groups were performed using one-way analysis of variance (ANOVA) with a Tukey’s multiple comparisons test. ∗*p* < 0.05; ∗∗*p* < 0.01; ∗∗∗*p* < 0.001; NS, not significant. Error bars in the graphical representations correspond to the means ± SDs.

## Supplemental information

Document S1. Figures S1–S13 and Table S1

Document S2. Article plus supplemental information
