# Supplementary material for: mRNA lipid-nanoparticle-mediated mitochondrial apoptosis augments adoptive T cell immunotherapy
Source: Cell Rep Med. 2026 Mar 30;7(4):102706. doi: 10.1016/j.xcrm.2026.102706 (PMC13130659; doi:10.1016/j.xcrm.2026.102706)
Supplement: Document S1. Figures S1–S13 and Table S1 [file mmc1.pdf]

Cell Reports Medicine, Volume *7*

Supplemental information

mRNA lipid-nanoparticle-mediated mitochondrial apoptosis augments adoptive T cell immunotherapy

Jiayan Fu, Yaoqi Liu, Zhenyu Zhong, Benyuan Cao, Luna Ran, Zijia Guo, Haiyang Dong, Nengcheng Bao, Rongqing Pan, Jinqiang Wang, Yuanhui Mao, and Yongfeng Jin

Supporting information

mRNA lipid nanoparticle-mediated mitochondrial apoptosis augments adoptive T cell immunotherapy

Jiayan Fu[1,2], Yaoqi Liu[1,2], Zhenyu Zhong[2], Benyuan Cao[1,2], Luna Ran[1,2], Zijia Guo[1,2], Haiyang Dong[1,2], Nengcheng Bao[1,2], Rongqing Pan[3], Jinqiang Wang[1], Yuanhui Mao[4*], Yongfeng Jin[1,2,5,6,7*]

This PDF file includes:

Tables S1

Figures S1 to S13

Table S1. Open reading frame for therapeutic mRNA designed in this study. Related to STAR Methods.

Open reading frame for mRNA design
**Puma (with Scaffold) ORF**<br>ATGATCCCCCGCGAGGAGCAGTGGGCGCGCGAGATCGGTGCACAACTGCGCAGAATGGCGGAT<br>GATCTGAATGCTCAGTATGAGCGTAGGGGACTGTCCGAGGCTAAACCAGCCACTCCTGAGATTC<br>AAGAAATTGTGGATAAAGTAAAACCTCAGCTGGAGGAGAAAACTAATGAAACATATGGGAAAC<br>TCGAAGCCGTGCAGTACAAGACGCAAGTTCTGGCGAGCACCAATTACTATATCAAGGTGCGCGC<br>AGGCGATAATAAATATATGCACCTGAAAGTATTTAACGGGCCGCCAGGGCAGAATGCAGATAG<br>AGTGCTGACAGGATATCAGGTCGATAAAAATAAGGACGATGAACTGACGGGTTTCGATTACAAA<br>GATGACGATGACAAATAA
**Bim (with Scaffold) ORF**<br>ATGATCCCCCGCGACATGAGACCCGAGATCTGGATCGCTCAGGAGCTGAGAAGAATCGGCGAC<br>GAGTTCAACGCTTACTACGCTAGAAGAGGACTGTCCGAGGCTAAACCAGCCACTCCTGAGATTC<br>AAGAAATTGTGGATAAAGTAAAACCTCAGCTGGAGGAGAAAACTAATGAAACATATGGGAAAC<br>TCGAAGCCGTGCAGTACAAGACGCAAGTTCTGGCGAGCACCAATTACTATATCAAGGTGCGCGC<br>AGGCGATAATAAATATATGCACCTGAAAGTATTTAACGGGCCGCCAGGGCAGAATGCAGATAG<br>AGTGCTGACAGGATATCAGGTCGATAAAAATAAGGACGATGAACTGACGGGTTTCGATTACAAA<br>GATGACGATGACAAATAA
**Bad (with Scaffold) ORF**<br>ATGATCCCCCGCAACCTGTGGGCTGCTCAGAGATACGGCAGAGAGCTGAGAAGAATGAGCGAC<br>GAGTTCGTCGACAGCTTCAAGAAGGGCGGACTGTCCGAGGCTAAACCAGCCACTCCTGAGATTC<br>AAGAAATTGTGGATAAAGTAAAACCTCAGCTGGAGGAGAAAACTAATGAAACATATGGGAAAC<br>TCGAAGCCGTGCAGTACAAGACGCAAGTTCTGGCGAGCACCAATTACTATATCAAGGTGCGCGC<br>AGGCGATAATAAATATATGCACCTGAAAGTATTTAACGGGCCGCCAGGGCAGAATGCAGATAG<br>AGTGCTGACAGGATATCAGGTCGATAAAAATAAGGACGATGAACTGACGGGTTTCGATTACAAA<br>GATGACGATGACAAATAA
**Noxa (with Scaffold) ORF**<br>ATGATCCCCCGCCCCGCTGAGCTGGAGGTCGAGTGCGCTACCCAGCTGAGAAGATTCGGCGACA<br>AGCTGAACTTCAGACAGAAGCTGCTGGGACTGTCCGAGGCTAAACCAGCCACTCCTGAGATTCA<br>AGAAATTGTGGATAAAGTAAAACCTCAGCTGGAGGAGAAAACTAATGAAACATATGGGAAACT<br>CGAAGCCGTGCAGTACAAGACGCAAGTTCTGGCGAGCACCAATTACTATATCAAGGTGCGCGCA<br>GGCGATAATAAATATATGCACCTGAAAGTATTTAACGGGCCGCCAGGGCAGAATGCAGATAGA<br>GTGCTGACAGGATATCAGGTCGATAAAAATAAGGACGATGAACTGACGGGTTTCGATTACAAAG<br>ATGACGATGACAAATAA
**Puma (without Scaffold) ORF**<br>ATGGAGGAGCAGTGGGCGCGCGAGATCGGTGCACAACTGCGCAGAATGGCGGATGATCTGAAT<br>GCTCAGTATGAGCGTAGGTAA
**Bim (without Scaffold) ORF**<br>ATGGACATGAGACCCGAGATCTGGATCGCTCAGGAGCTGAGAAGAATCGGCGACGAGTTCAAC<br>GCTTACTACGCTAGAAGATAA
**Bad (without Scaffold) ORF**<br>ATGAACCTGTGGGCTGCTCAGAGATACGGCAGAGAGCTGAGAAGAATGAGCGACGAGTTCGTC<br>GACAGCTTCAAGAAGGGCTAA

Noxa (without Scaffold) ORF

ATGCCCGCTGAGCTGGAGGTCGAGTGCGCTACCCAGCTGAGAAGATTCGGCGACAAGCTGAACT
TCAGACAGAAGCTGCTGTAA

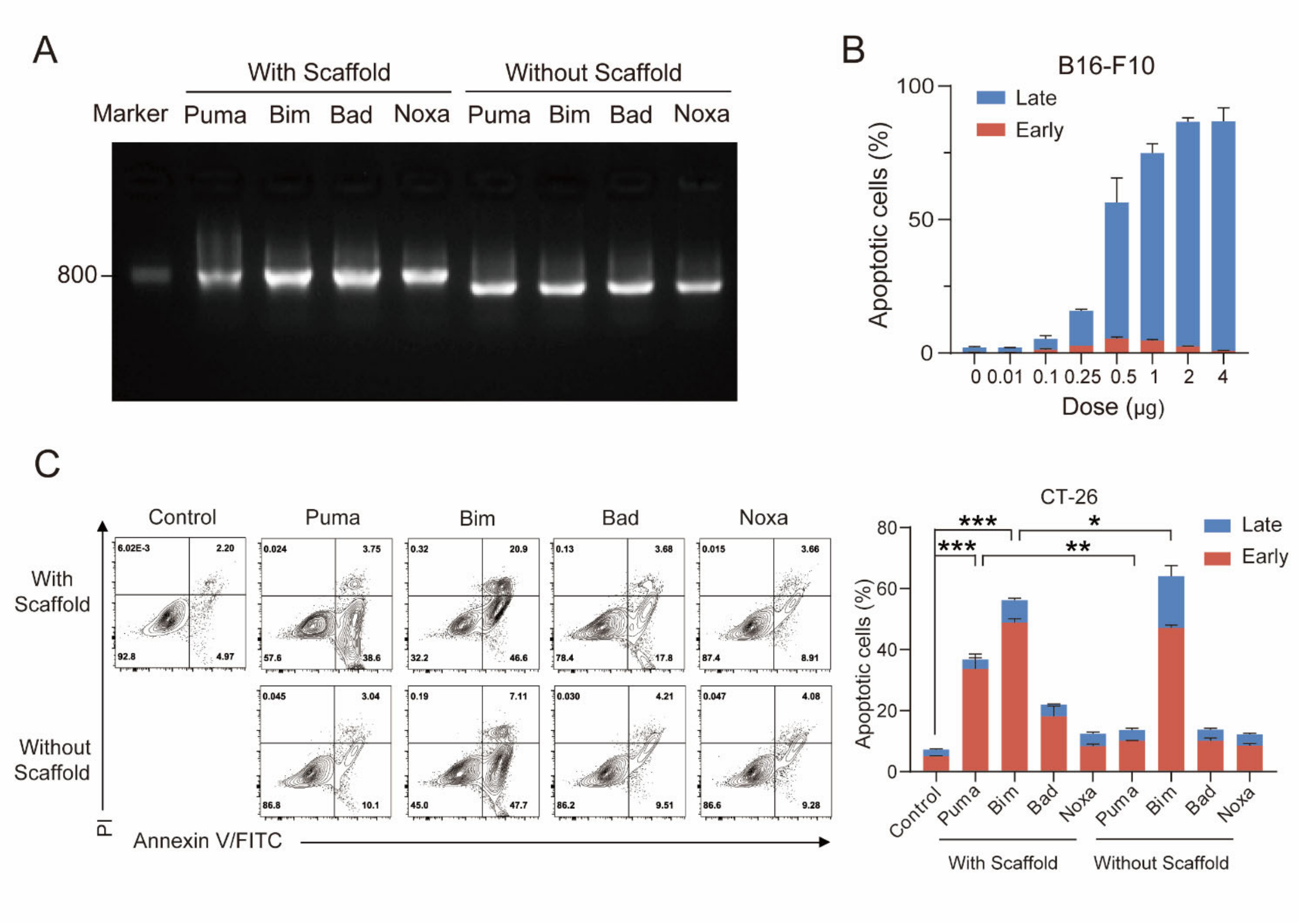

Figure S1. Characterization of mRNA/LNP encoding distinct BH3 domains. Related to Figure 1.

(A) RNA gel imaging for *in vitro* transcribed mRNA encoding distinct BH3 domains (Puma, Bim, Bad, Noxa) with or without aptamer scaffold.

(B) Flow cytometric analysis of apoptosis in B16-F10 tumor cells after treatment with various doses of mBH3@NPs (using mBim@NPs as an example) for 24 h.

(C) Flow cytometric analysis and quantification of apoptosis in CT-26 tumor cells after treatment with mBH3@NPs encoding distinct BH3 domains, with or without scaffold for 8 h (n = 3).

Two-tailed unpaired Student's t test. All data are presented as the mean ± SD. *P < 0.05; **P < 0.01; ***P < 0.001; NS, not significant.

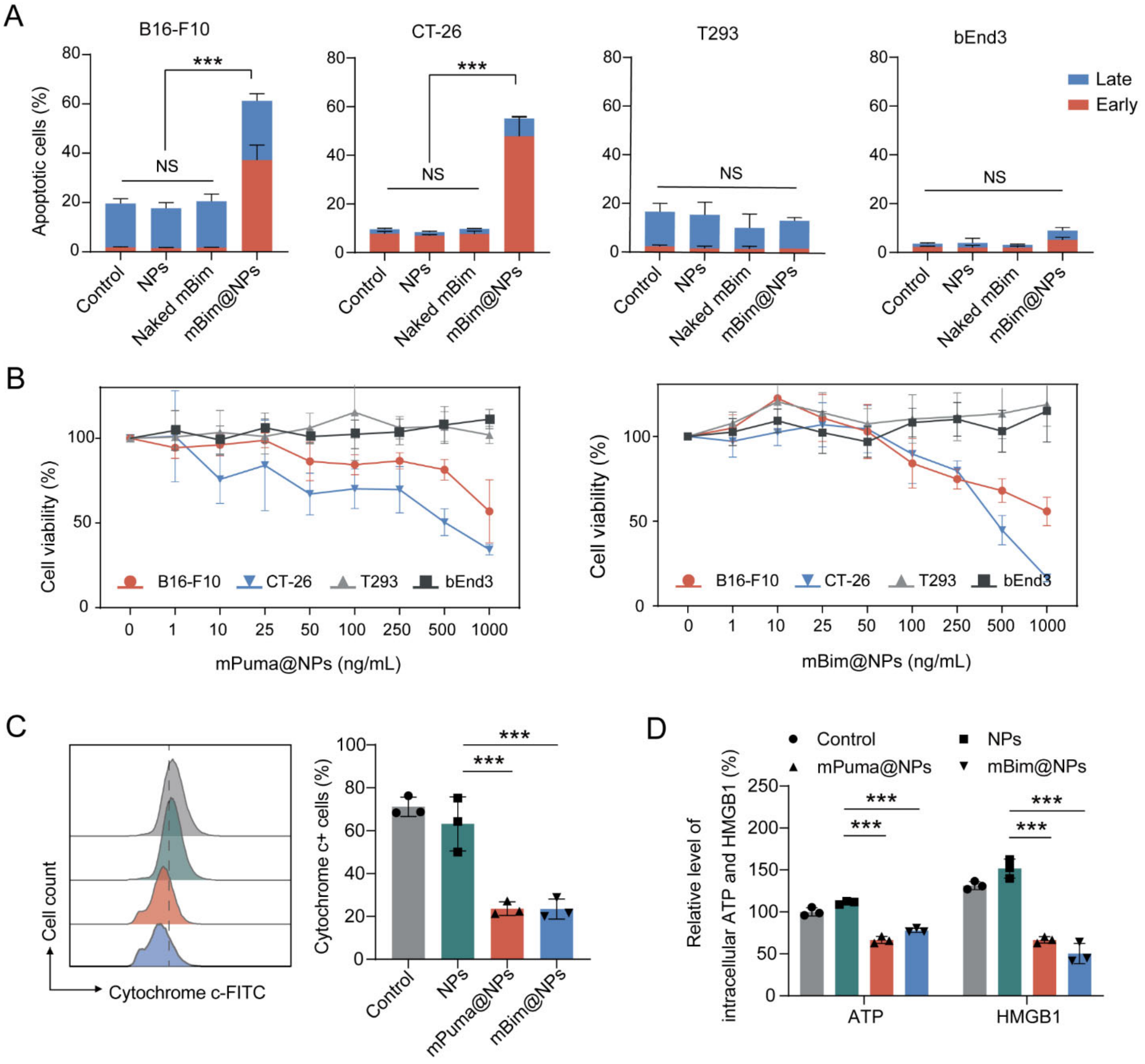

Figure S2. mBH3@NPs preferentially induced mitochondrial apoptosis and immunogenic cell death in cancer cells. Related to Figure 2.

(A) Flow cytometry analysis of annexin V and propidium iodide-positive cells in cancer and non-cancer cell lines treated with Control, NPs, naked mBim, and mBim@NPs for 12 h ($n = 3$).

(B) Cell viability of cancer cell lines (B16-F10, CT-26) and non-cancer cell lines (bEnd3, T293) after 12 h incubation with mPuma@NPs and mBim@NPs.

(C) Flow cytometry analysis of intracellular cytochrome c levels in B16-F10 cells following 12 h treatment with PBS (Control), NPs, mPuma@NPs, and mBim@NPs. Cells were permeabilized prior to staining with a FITC-conjugated anti-cytochrome c antibody to assess the cytochrome c retention.

(D) Intracellular ATP and HMGB1 levels were analyzed by ELISA in B16-F10 cells after 12 h incubation with PBS (control), NPs, mPuma@NPs, and mBim@NPs (n = 3).

One-way ANOVA with Tukey's multiple comparisons test was used for all statistical analyses. Data are presented as the mean ± SD. $*P < 0.05$; $**P < 0.01$; $***P < 0.001$; NS, not significant.

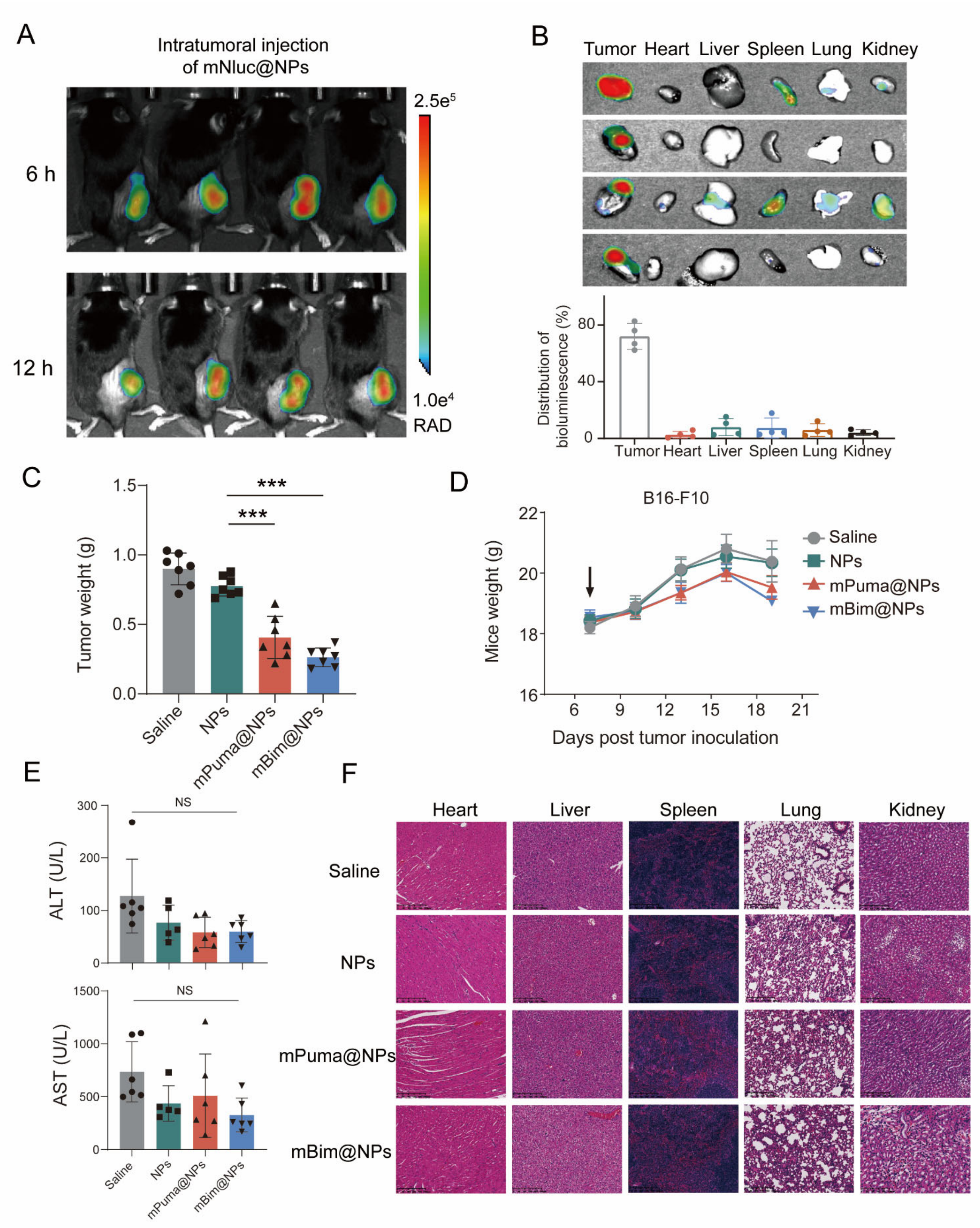

Figure S3. Biodistribution and biosafety of mBH3@NPs. Related to Figure 3.

(A) *In vivo* bioluminescence imaging of mice at 6 and 12 hours after intratumoral administration of mNluc@NPs in melanoma model (n = 4).

(B) *Ex vivo* bioluminescence imaging of tumors and major organs harvested at 12 hours post-injection

(n = 4). Quantitative analysis showing the percentage of total signal distributed in tumor and each organ.

(C) The tumor weight in the B16-F10 melanoma model (n = 7).

(D) The mice weight in the B16-F10 melanoma model (n = 7).

(E) AST levels and ALT levels of B16-F10-bearing mice after intratumoral injection of saline, NPs, mPuma@NPs, and mBim@NPs respectively. AST, Aspartate transaminase. ALT, alanine aminotransferase.

(F) The major organs (heart, liver, spleen, lung, and kidney) were collected at day 19 and analyzed by H&E staining to evaluate the biosafety. Scale bar, 200 μm.

One-way ANOVA with Tukey's multiple comparisons test was used for all statistical analyses. Data are presented as the mean ± SD. *P < 0.05; **P < 0.01; ***P < 0.001; NS, not significant.

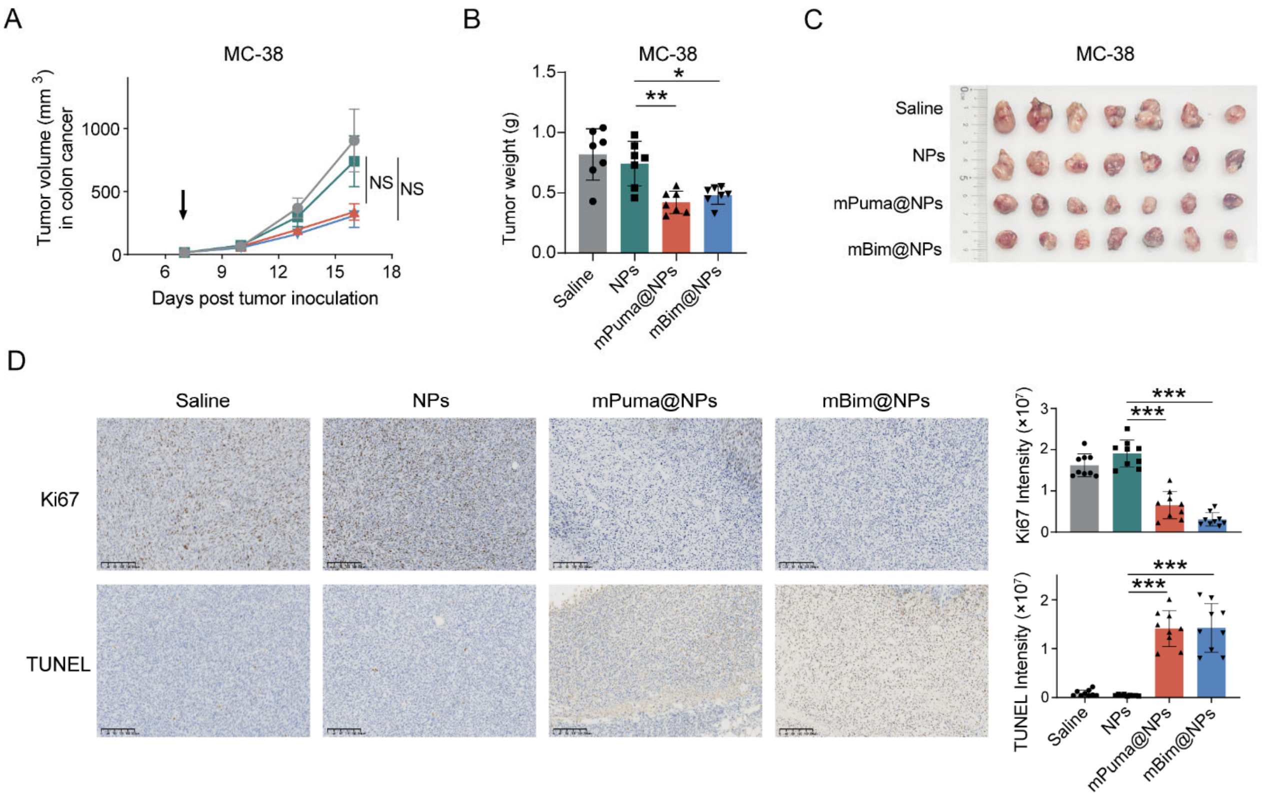

Figure S4. The therapeutic effects of mBH3@NPs in MC-38 model. Related to Figure 3.

(A) The average tumor volume curves for mice treated in colon cancer MC-38 model (n = 7).

(B) The tumor weight in colon cancer MC-38 model (n = 7).

(C) The tumor images for mice treated in colon cancer MC-38 model (n = 7).

(D) Representative immunohistochemistry staining and quantification of Ki-67 and TUNEL of tumor, scale bar, 200 μm.

One-way ANOVA with Tukey's multiple comparisons test was used for all statistical analyses. Data are presented as the mean ± SD. *P < 0.05; **P < 0.01; ***P < 0.001; NS, not significant.

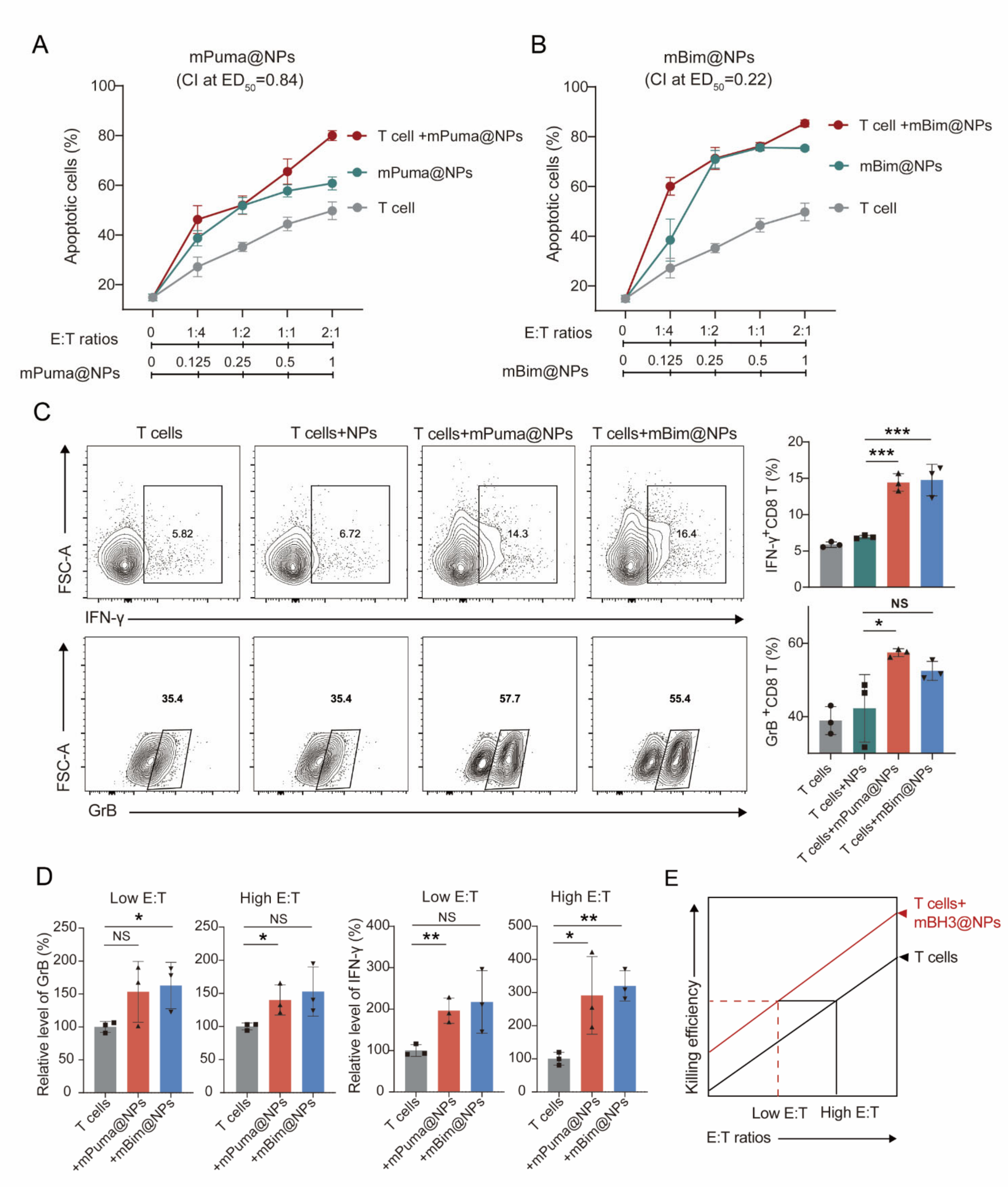

Figure S5. mBH3@NPs synergized with T cells in killing effects *in vitro*. Related to Figure 4.

(A) Synergistic cytotoxicity of mPuma@NPs and OT-1 T cells against B16-OVA cells, measured by Annexin V/PI staining. Cytotoxicity is shown as the mean of three different assays. Green, gray, and red lines indicate cytotoxic effects of mPuma@NPs, T cells alone, and T cells+mPuma@NPs, respectively. The combination index at the ED_{50} was calculated using CompuSyn (n = 3).

(B) Synergistic cytotoxicity of mBim@NPs and OT-1 T cells against B16-OVA cells, measured by

flow by Annexin V/PI staining.

(C) Representative flow cytometry graphs and quantification of $IFN^{+}CD8^{+}$ T cells and $GrB^{+}CD8^{+}$ T cells. B16-OVA cells were treated with PBS (Control), NPs, mPuma@NPs, and mBim@NPs, followed by co-culture with pre-activated OT-1 T cells at E:T ratio of 1:2 for 24 h. $CD8^{+}$ T cells were harvested and analyzed by flow cytometry ($n = 3$).

(D) GrB and IFN-γ secretion levels in the cell supernatant following treatments with PBS, mPuma@NPs, and mBim@NPs at both low and high E:T ratios, analyzed by ELISA ($n = 3$).

(E) Schematic model illustrating how mBH3@NPs synergize with T cell-mediated killing.

One-way ANOVA with Tukey's multiple comparisons test was used for all statistical analyses. Data are presented as the mean ± SD. *$P < 0.05$; **$P < 0.01$; ***$P < 0.001$; NS, not significant.

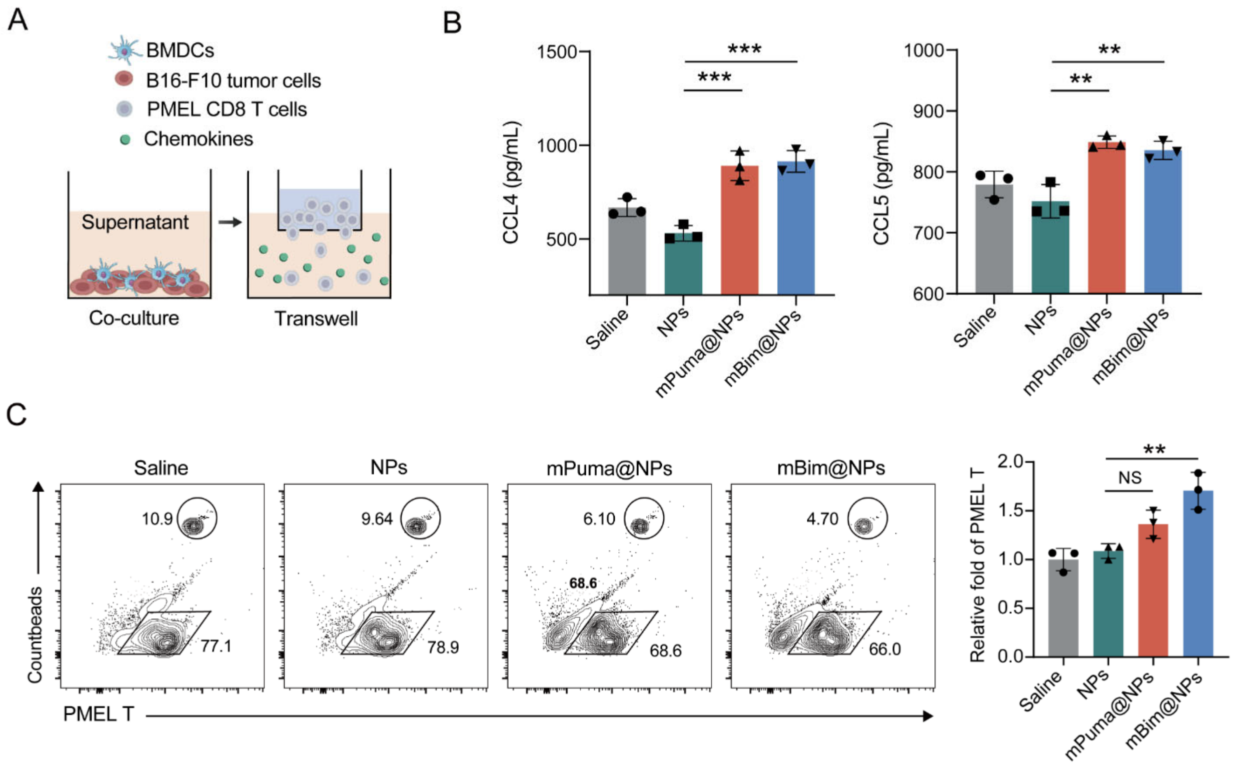

Figure S6. mBH3@NPs promoted the recruitment of adoptively transferred T cells by secreting key chemokines. Related to Figure 5.

(A) Schematic illustration of the transwell migration assay using co-culture supernatant and pre-activated PMEL T cells. The co-culture was performed between mBH3@NPs-treated B16-F10 tumor cells and BMDCs for 18 h.

(B) Expression levels of chemokines CCL4 and CCL5 in the cell supernatant after different treatments ($n = 3$).

(C) Representative flow cytometry graphs and quantification of relative T cell infiltration, calculated as the ratio of PMEL T cells in the bottom well to counting beads ($n = 3$).

One-way ANOVA with Tukey's multiple comparisons test was used for all statistical analyses. Data are presented as the mean ± SD. *$P < 0.05$; **$P < 0.01$; ***$P < 0.001$; NS, not significant.

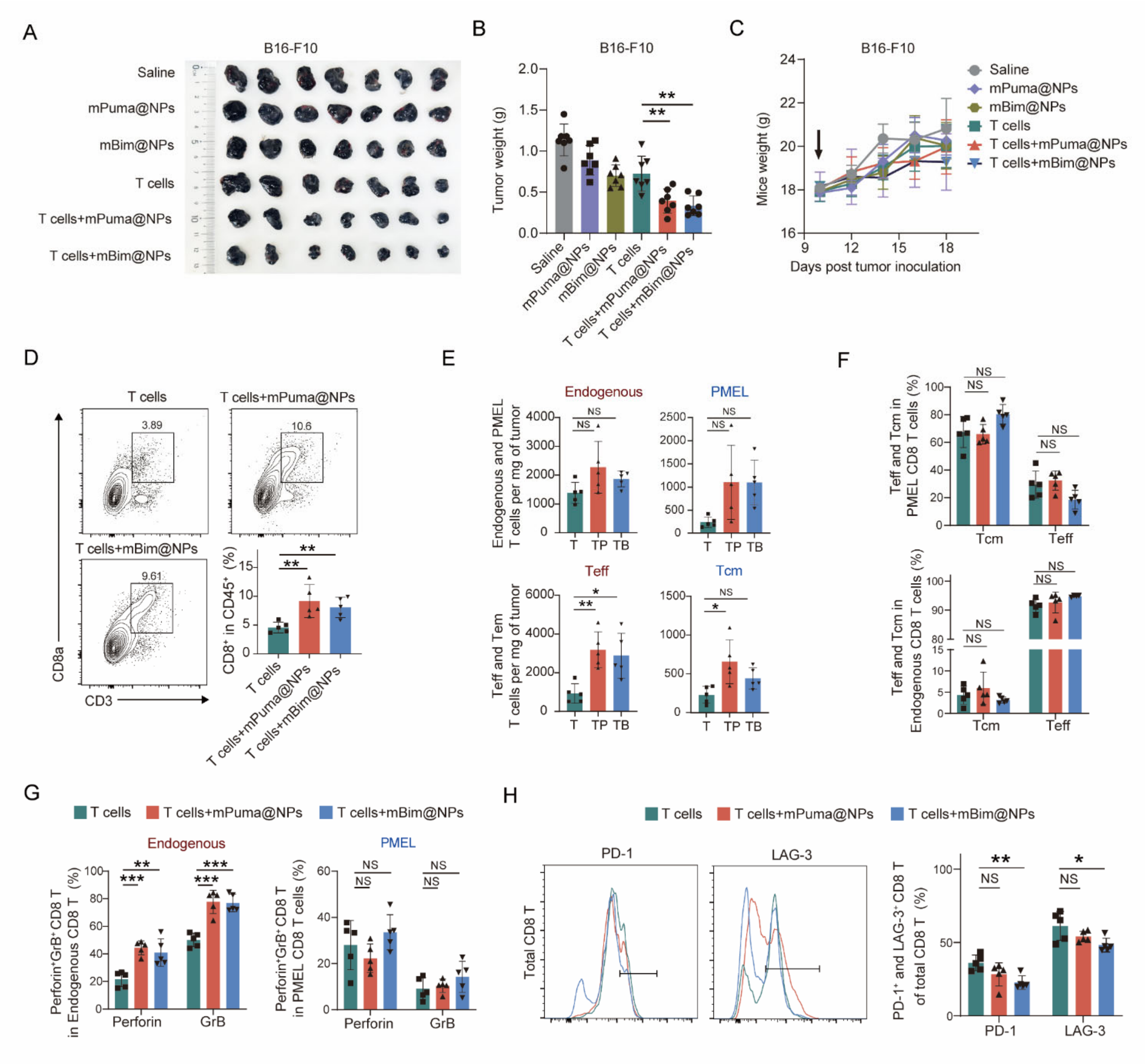

Figure S7. mBH3@NPs combined with adoptive T cell therapy enhanced the polyfunctionality of both endogenous and transferred effector T cells in B16-F10 melanoma model. Related to Figure 6.

(A) The tumor image for mice treated in B16-F10 melanoma model (*n* = 7).

(B) The tumor weight in B16-F10 melanoma model (*n* = 7).

(C) The mice weight in B16-F10 melanoma model (*n* = 7).

(D) Representative flow cytometry plot and quantification of infiltrating $CD8^+$ T cells from tumors (*n* = 5).

(E) Quantification of tumor-infiltrating $CD8^+$ T cells, including endogenous (CD90.2) $CD8^+$ T cells, transferred PMEL (CD90.1) $CD8^+$ T cells, T_{eff}, and T_{em} by flow cytometry, and normalized to tumor mass (cell per mg of tumor) (*n* = 5).

(F) Quantification of the expression level of CD44 and CD62L in both PMEL $CD8^+$ T cells and endogenous $CD8^+$ T cells from tumors (*n* = 5).

(G) Flow cytometry quantification of the expression level of Perforin and GrB in both PMEL $CD8^+$ T cells and endogenous $CD8^+$ T cells from tumors ($n = 5$).

(H) Representative flow cytometry plot and quantification of the expression level of PD-1 and LAG-3 in total $CD8^+$ T cells from tumors ($n = 5$).

One-way ANOVA with Tukey's multiple comparisons test was used for all statistical analyses. Data are presented as the mean ± SD. *$P < 0.05$; **$P < 0.01$; ***$P < 0.001$; NS, not significant.

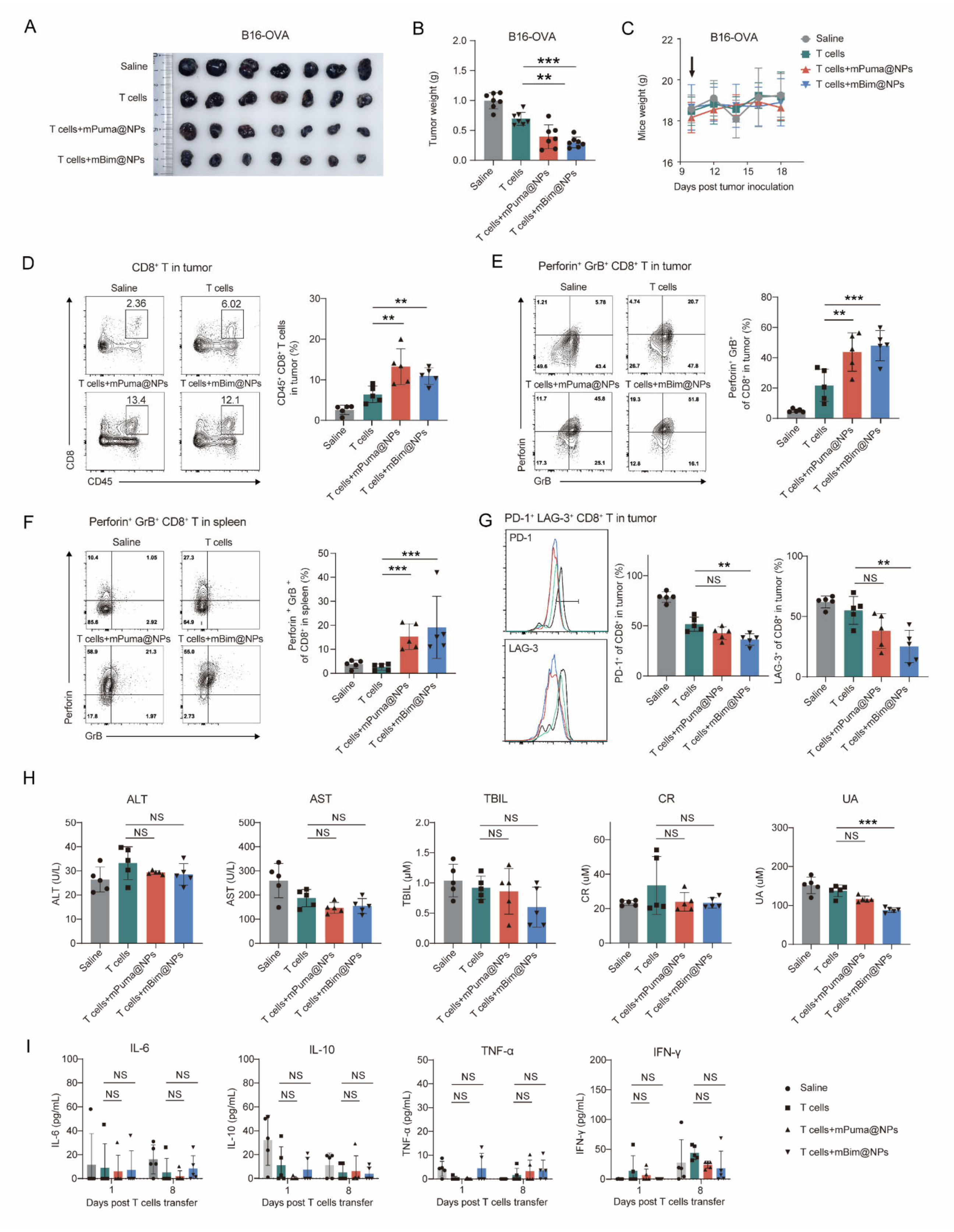

Figure S8. mBH3@NPs combined with adoptive T cell therapy improved the therapeutic effect in B16F10-OVA melanoma model. Related to Figure 6.

(A) The tumor image for mice treated in B16-OVA melanoma model (*n* = 7).

(B) The tumor weight in B16-OVA melanoma model (*n* = 7).

(C) The mice weight in B16-OVA melanoma model (*n* = 7).

(D) Representative flow cytometry plot and quantification of infiltrating $CD8^+$ T cells from tumors (*n* = 5).

(E and F) Representative flow cytometry plot and quantification of the expression level of GrB and Perforin in total $CD8^+$ T cells from tumors (E) and spleens (F) (*n* = 5).

(G) Representative flow cytometry plot and quantification of the expression level of PD-1 and LAG-3 in total $CD8^+$ T cells from the tumors (*n* = 5).

(H) ALT, AST, TBIL, CR, and UA levels of B16-OVA-bearing mice after treatment of saline, T cells alone, T cells+mPuma@NPs, and T cells+mBim@NPs. ALT, alanine aminotransferase. AST, aspartate transaminase. TBIL, total bilirubin. CR, creatinine. UA, urea (*n* = 5).

(I) Serum cytokine levels of key CRS-associated cytokines (IL-6, IL-10, TNF-α, and IFN-γ) of B16-OVA-bearing mice after tratement of saline, T cells alone, T cells+mPuma@NPs, and T cells+mBim@NPs (*n* = 5).

One-way ANOVA with Tukey’s multiple comparisons test was used for all statistical analyses. Data are presented as the mean ± SD. *$P < 0.05$; **$P < 0.01$; ***$P < 0.001$; NS, not significant.

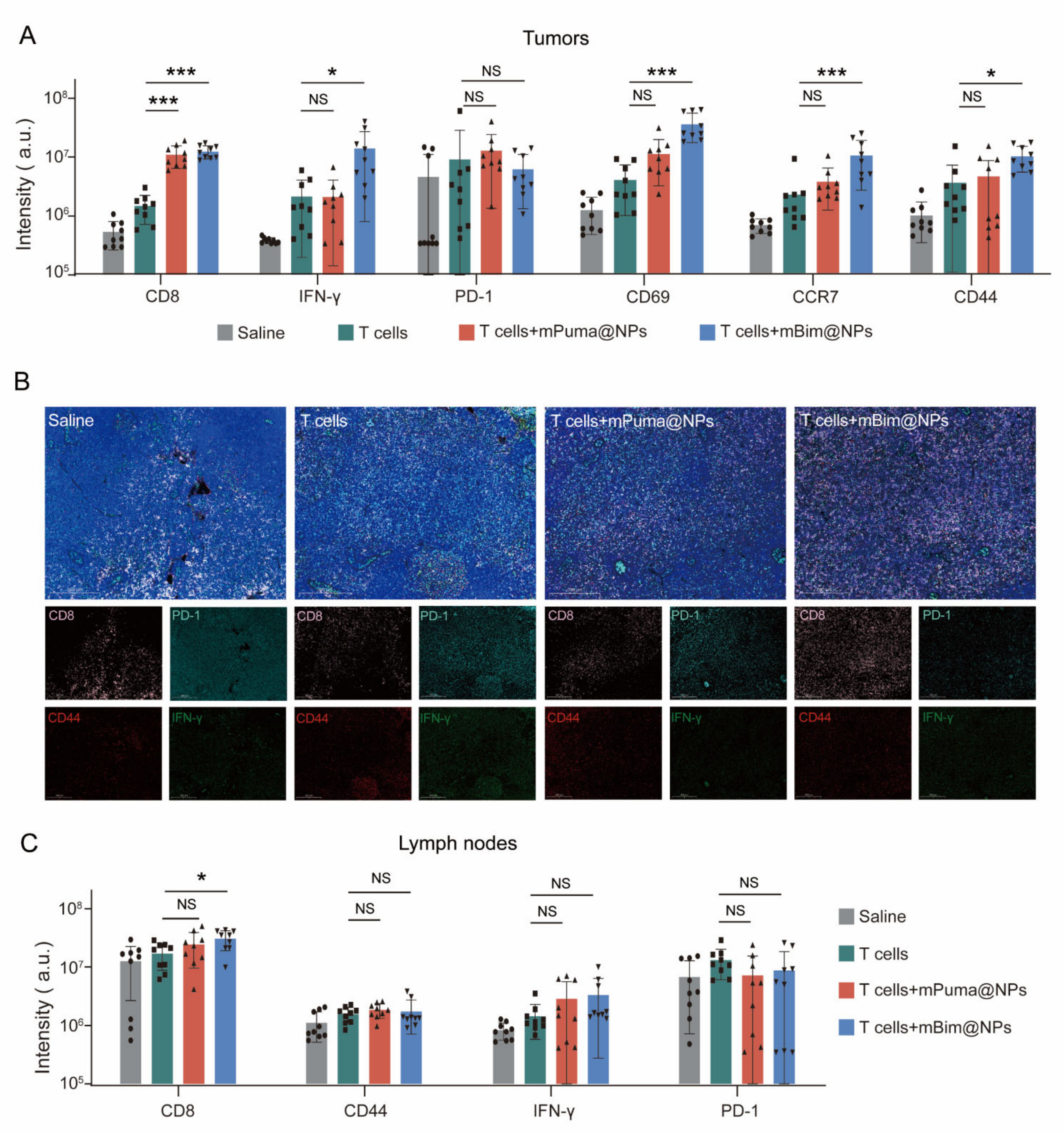

Figure S9. mBH3@NPs combined with adoptive T cell therapy enhanced effector T cell polyfunctionality. Related to Figure 6.

(A) Quantification of CD8, IFN-γ, PD-1, CD69, CCR7, and CD44 in tumor tissues shown in upper panel of Figure 6J.

(B) Representative immunofluorescence images of the draining lymph nodes for analyzing the exhaustion (PD-1^+, cyan), memory ($CD44^+$, red), and effector (IFN-γ^+, green) function of $CD8^+$ T cells ($CD8^+$, pink). Scale bars, 200 μm ($n = 3$).

(C) Quantification of CD8, CD44, IFN-γ, and PD-1 in lymph nodes shown in Figure S9B.

One-way ANOVA with Tukey's multiple comparisons test was used for all statistical analyses. Data

are presented as the mean ± SD. *$P < 0.05$; **$P < 0.01$; ***$P < 0.001$; NS, not significant.

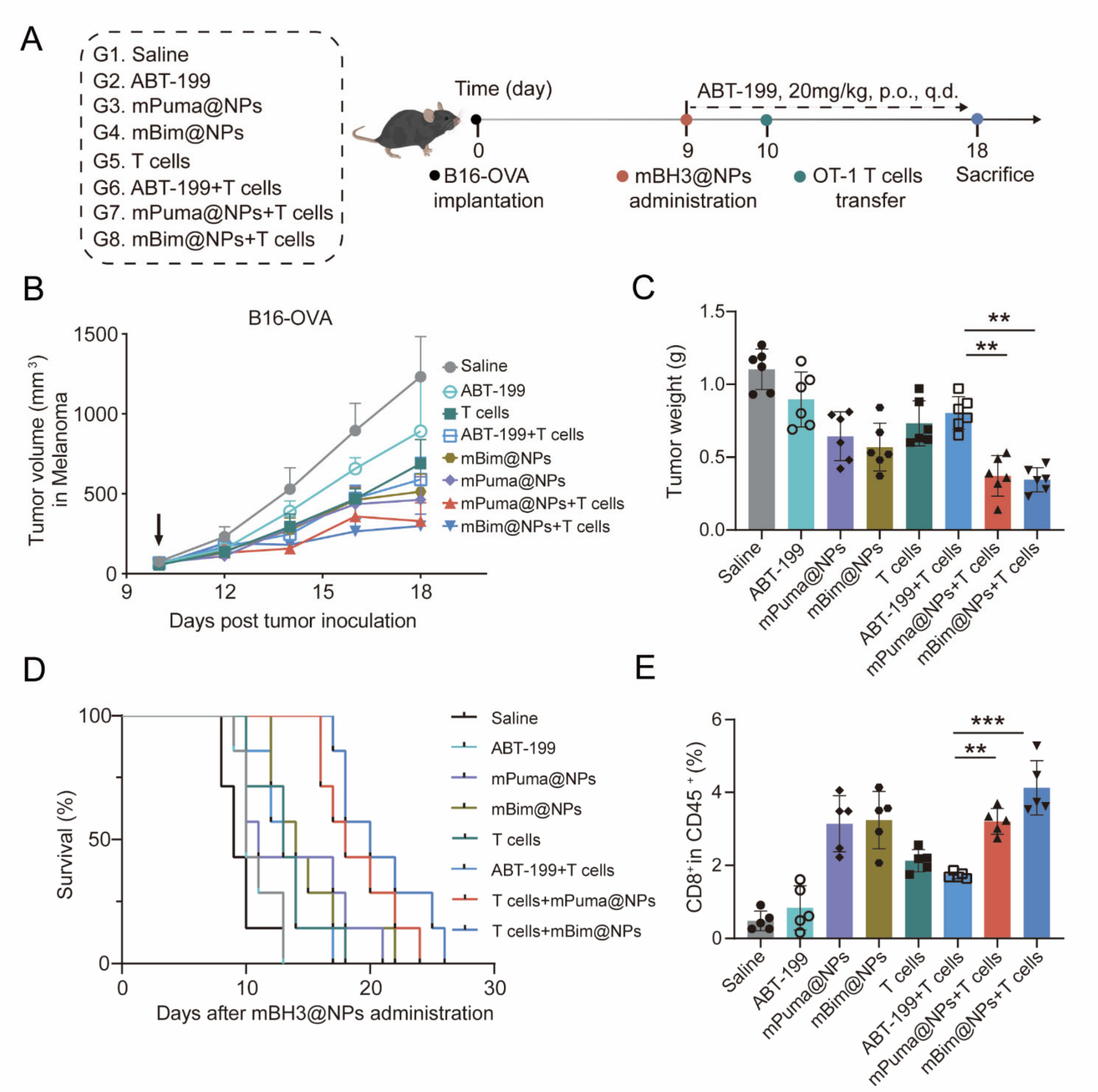

Figure S10. Efficacy of mBH3@NPs and the BH3 mimetic ABT-199 in combination with adoptive T cell therapy. Related to Figure 6.

(A) Schematic diagram of experimental design and timeline for the head-to-head comparison of mBH3@NPs and the clinical BH3 mimetic ABT-199 in combination with adoptive transferred T cells from OT-1 mice in B16-OVA tumor-bearing mice.

(B) The average tumor volume curves for mice treated in B16-OVA tumor-bearing mice model (n = 7).

(C) The tumor weight in B16-OVA melanoma model (n = 6).

(D) Survival analysis for mice treated in B16-OVA tumor-bearing mice model ($n = 7$). Mice were humanely euthanized when tumor volume reached the predefined ethical endpoint (15 mm×15 mm). The x-axis indicates days post mBH3@NPs administration.

(E) Quantification of infiltrating $CD8^+$ T cells from tumors in each groups ($n = 5$).

One-way ANOVA with Tukey's multiple comparisons test was used for all statistical analyses. Data are presented as the mean ± SD. *$P < 0.05$; **$P < 0.01$; ***$P < 0.001$; NS, not significant.

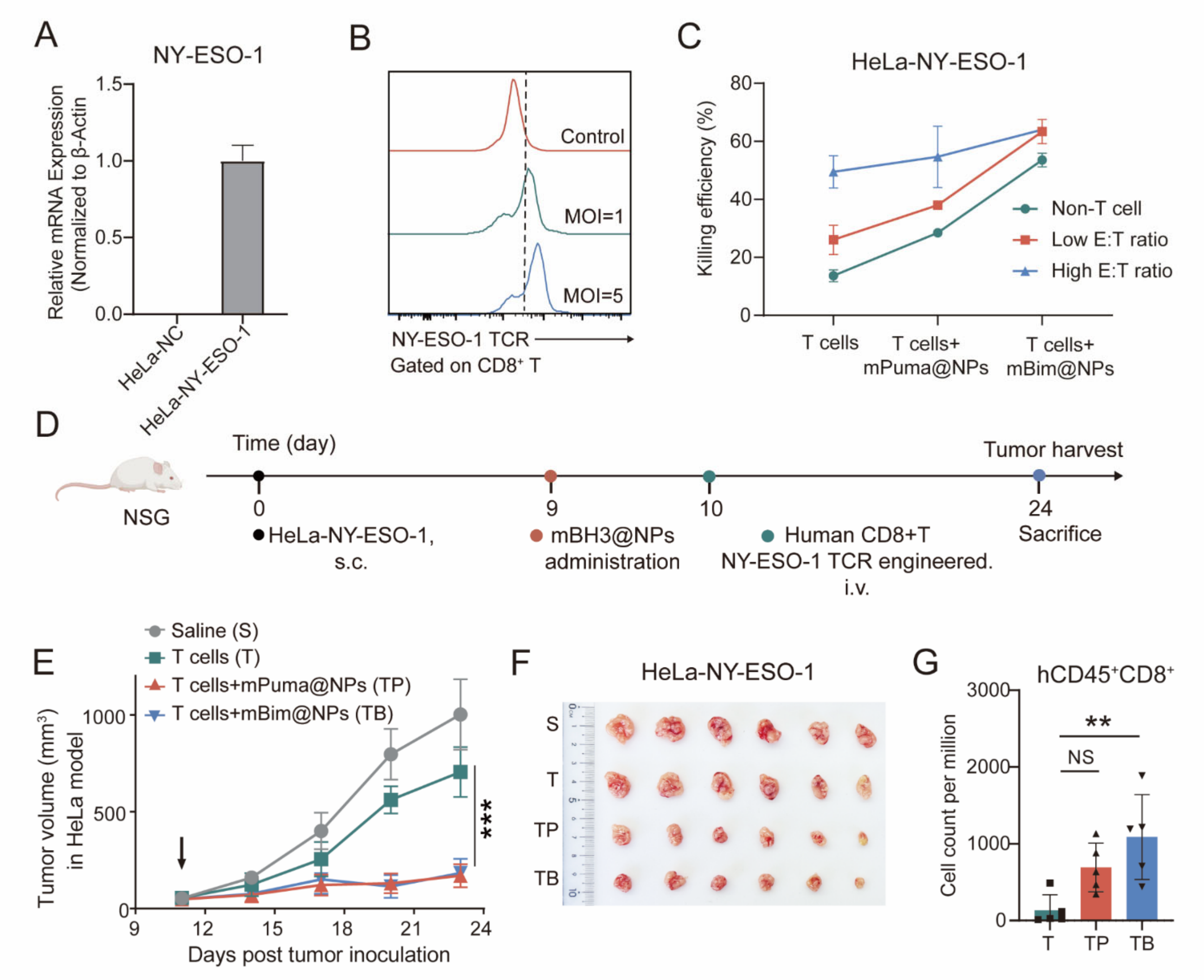

Figure S11. Enhanced therapeutic effect of combined mBH3@NPs and adoptive T cell therapy in a humanized xenograft model. Related to Figure 6.

(A) qRT-PCR validation of NY-ESO-1 overexpression in engineered HeLa cell lines (n = 3).

(B) $CD8^+$ T cells derived from PBMC positive for NY-ESO-1 TCR after 48 hours of lentivirus infection at different MOIs.

(C) Quantification of NY-ESO-1 TCR^+ $CD8^+$T cell-mediated killing of HeLa-NY-ESO-1 cells were pre-treated with PBS, mPuma@NPs, and mBim@NPs and co-cultured at indicated E:T ratios (n = 3).

(D) Experimental timeline for mBH3@NPs administration combined with adoptive transfered T cells in HeLa-NY-ESO-1 humanized xenograft model.

(E) The average tumor volume curves for mice treated in HeLa-NY-ESO-1 tumor-bearing mice model (n = 7).

(F) Representative tumor image for mice treated in HeLa-NY-ESO-1 humanized xenograft model (n = 6).

(G) Quantification of infiltrating $hCD45^+$ $CD8^+$ T cells from tumors in each groups (n = 5).

One-way ANOVA with Tukey's multiple comparisons test was used for all statistical analyses. Data are presented as the mean ± SD. *$P < 0.05$; **$P < 0.01$; ***$P < 0.001$; NS, not significant.

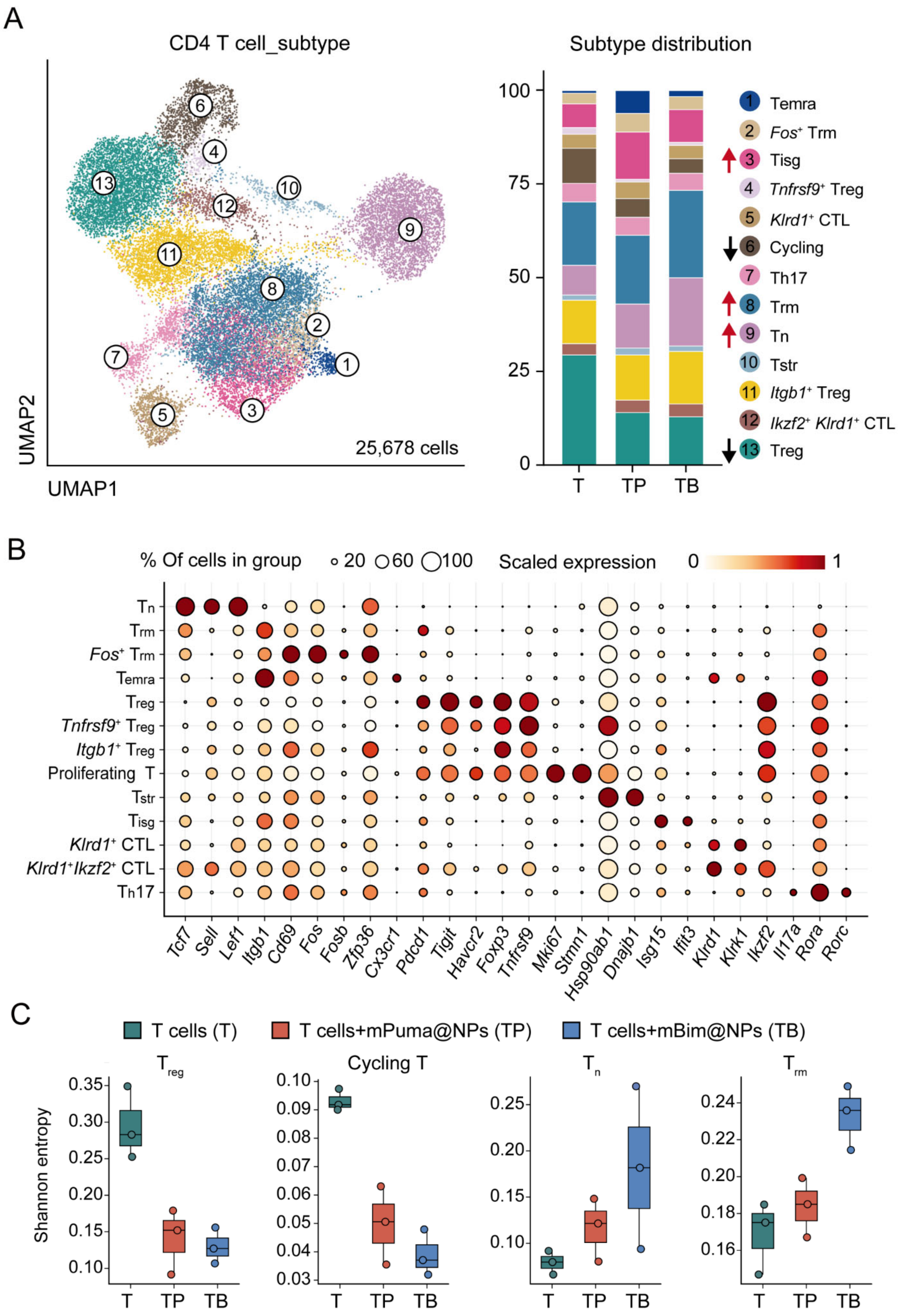
A
CD4 T cell_subtype
UMAP2
UMAP1
25,678 cells
Subtype distribution
100
75
50
25
0
T
TP
TB
1 Temra
2 Fos+ Trm
3 Tisg
4 Tnfrsf9+ Treg
5 Klrd1+ CTL
6 Cycling
7 Th17
8 Trm
9 Tn
10 Tstr
11 Itgb1+ Treg
12 Ikzf2+ Klrd1+ CTL
13 Treg
B
% Of cells in group
20
60
100
Scaled expression
0
1
Tn
Trm
Fos+ Trm
Temra
Treg
Tnfrsf9+ Treg
Itgb1+ Treg
Proliferating T
Tstr
Tisg
Klrd1+ CTL
Klrd1+Ikzf2+ CTL
Th17
Tcf7
Sell
Lef1
Itgb1
Cd69
Fos
Fosb
Zfp36
Cx3cr1
Pdcd1
Tigit
Havcr2
Foxp3
Tnfrsf9
Mki67
Stmn1
Hsp90ab1
Dnajb1
Isg15
Ifit3
Klrd1
Klrk1
Ikzf2
Il17a
Rora
Rorc
C
T cells (T)
T cells+mPuma@NPs (TP)
T cells+mBim@NPs (TB)
Shannon entropy
Treg
Cycling T
Tn
Trm
0.35
0.30
0.25
0.20
0.15
0.10
0.10
0.09
0.08
0.07
0.06
0.05
0.04
0.03
0.25
0.20
0.15
0.10
0.24
0.22
0.20
0.18
0.16
T
TP
TB

Figure S12. Transcriptional landscape of $CD4^+$ T cells in TME by scRNA-seq. Related to Figure 7.

(A) UMAP representation and distribution of 25,678 cells of the $CD4^+$ T cell atlas in each experimental condition, colored by 13 $CD4^+$ T cell subtypes annotated in this study.

(B) A dot plot showing the expression levels of marker genes in each $CD4^+$ T cell subtype. The color of the dots indicates the average scaled expression level, and the size of the dots indicates the percentage of cells expressing the gene in each subtype.

(C) Shannon entropy of the T_{ex}, cycling T, T_{naive}, T_{rm} $CD4^+$ T subsets across different treatment groups (T: ACT monotherapy; TP: ACT + mPuma@NPs; TB: ACT + mBim@NPs).

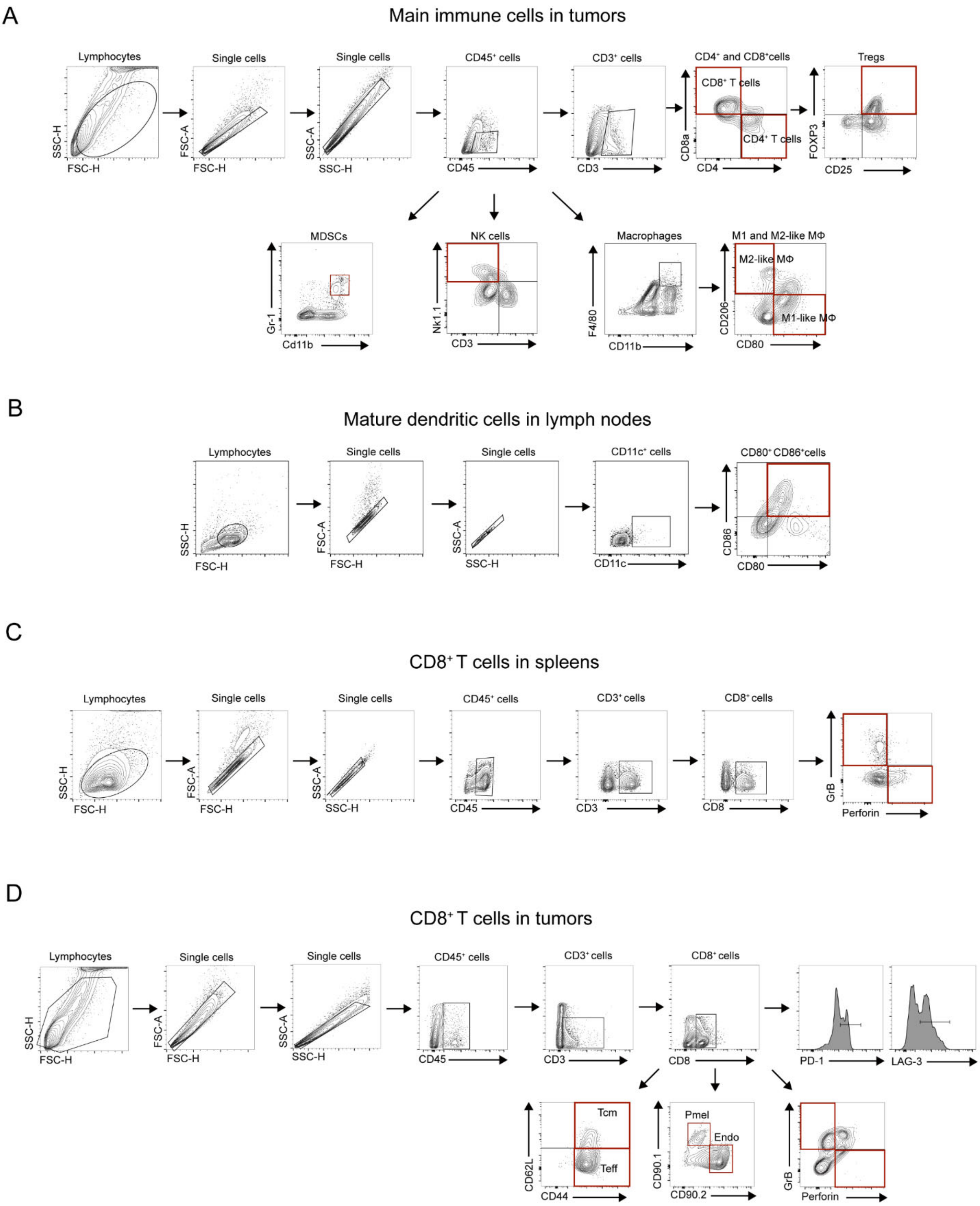

Figure S13. Gating strategies for main immune cells in tumors (A), mature dendritic cells in lymph nodes (B), CD8$^+$ T cells in spleens (C) and tumors (D). Related to Figures 3 and 6.
